# Acetylcholine and Prolactin, Other Factors in Breast Cancer

**DOI:** 10.3390/ijms27209216

**Published:** 2026-10-17

**Authors:** Gloria M. Calaf, Cristian Andrade-Madrigal, Leodan A. Crispin, Victor H. Miranda, Francisco Aguayo

**Affiliations:** 1 Instituto de Alta Investigación, Universidad de Tarapacá, Arica 1000000, Chile; kcrispi@gestion.uta.cl (L.A.C.); victor.miranda.pena@alumnos.uta.cl (V.H.M.); 2 Laboratorio de Oncovirología, Departamento de Ciencias Biomédicas, Facultad de Medicina, Universidad de Tarapacá, Arica 1000000, Chile; cristian.andradem@gmail.com (C.A.-M.); fraguayog@academicos.uta.cl (F.A.)

**Keywords:** acetylcholine, prolactin, breast cancer, STAT3, AKT, ERK, PRLR, ACHE, kinase arrays

## Abstract

Acetylcholine (ACh) and prolactin (PRL) are increasingly recognized as additional factors potentially influencing breast cancer biology. Their effects may depend on tumor-cell context and on the molecular signaling state of individual breast cancer subtypes. This study aimed to evaluate phosphorylation responses to ACh and PRL and examine transcriptomic associations involving related signaling genes. MCF7, T47D, and MDA-MB-231 breast cancer cell lines were treated with ACh, PRL, or combined ACh + PRL for 4 h. Significant changes in selected phosphoproteins were summarized for proteins related to AKT–S6 kinase, EGFR–ERK, TP53–Src/PLC-γ1, and STAT3–JUN signaling. Transcriptomic analyses evaluated associations between acetylcholinesterase (*ACHE*) or prolactin receptor (*PRLR*) expression and selected signaling-related genes in breast cancer cohorts, including subtype-specific analyses, tumor-versus-normal comparisons, and overall-survival associations. ACh and PRL produced distinct, cell-line-specific phosphorylation patterns. The MCF7 cell line showed increased AKT, p70S6K, and p53 phosphorylation after ACh, whereas combined treatment increased AKT and STAT3 phosphorylation. The T47D cell line showed a mixed response, with combined treatment increasing p70S6K, PLC-γ1, and Src phosphorylation but reducing AKT, ERK1/2, and c-Jun phosphorylation. The MDA-MB-231 cell line showed the broadest response, with combined ACh + PRL increasing AKT, RSK, STAT3, and c-Jun phosphorylation. Transcriptomic analyses identified subtype-dependent associations of *ACHE* and *PRLR* with genes encoding AKT, S6 kinase, ERK, Src/PLC-γ1, STAT3, and JUN-related proteins. Several genes also showed differential tumor-versus-normal expression or survival associations. ACh and PRL are associated with context-dependent signaling responses in breast cancer. Experimental phosphoprotein changes and tumor transcriptomic associations converged on related signaling modules but represented different molecular levels and did not establish complete pathway activation or direct causality. These findings support further investigation of ACh–PRL interactions and their potential contribution to breast cancer biology.

## 1. Introduction

Breast cancer is a global public health problem and the most prevalent cancer type affecting women worldwide, with about 2.4 million new cases annually and approximately 694,000 deaths in 2024 [1]. Breast cancer is a heterogeneous disease characterized by the unregulated proliferation of epithelial cells in lactiferous ducts or the lobules of the mammary gland [2].

The mammary gland is not only exposed to endogenous hormones but also to environmental agents capable of modifying its differentiation program, including organophosphorus pesticides (OPs) [3]. OPs inhibit acetylcholinesterase (ACHE), the enzyme responsible for acetylcholine (ACh) hydrolysis at cholinergic synapses [4], and epidemiological studies have recognized important links between numerous environmental pollutants and breast cancer progression [5].

ACh was the first neurotransmitter to be characterized, following the demonstration of chemical neurotransmission by Loewi in 1921 and the pharmacological and chemical work of Dale and co-workers [6]. It exerts not only synaptic functions but also autocrine and paracrine actions that promote cell proliferation [7,8] and inhibit apoptosis through acetylcholine receptors [9,10]. ACh can activate ERα through a ligand-independent mechanism involving ERK1/2-mediated phosphorylation at Ser118 and Ser104/106, promoting nuclear translocation and anchorage-independent growth in ERα-positive breast cancer cell lines [11].

Prolactin (PRL) is a 199-amino-acid polypeptide hormone synthesized primarily by lactotroph cells of the anterior pituitary that acts synergistically with estrogen and progesterone to promote mammary gland development and differentiation [12]. Beyond lactation, PRL exerts diverse biological actions, including cell differentiation, immune modulation, and adipocyte regulation [13]. Epidemiological studies have reported a positive association between serum PRL levels and breast cancer risk, particularly in ERα-positive and invasive tumors [14,15]. PRLR signaling has consequently been proposed as a therapeutic target and has been linked to endocrine therapy resistance [16].

Global gene expression analyses have defined five main intrinsic breast cancer subtypes: basal-like, luminal A, luminal B, HER2-enriched, and normal breast-like, to which the claudin-low group was subsequently added [17,18]. Estrogen receptor-α (ERα)-positive tumors represent nearly 80% of all breast cancer cases, reflecting a high dependence on estrogen signaling for tumor growth [19,20]. Incidence varies across populations, with age, family history, and genetic mutations as established risk factors [21,22,23].

ACh and PRL therefore converge on overlapping intracellular cascades, PI3K/AKT, RAS/MAPK, and STAT, yet they have almost always been studied in isolation, and it is not known whether their combination reproduces, amplifies, or opposes the effects of either mediator alone. The present study addresses this gap in two complementary steps. First, the individual and combined effects of ACh and PRL on 37 phosphorylation sites were profiled in three breast cancer cell lines representing distinct molecular subtypes such as the ERα-positive luminal models (MCF7, T47D cell lines) and a triple-negative line (MDA-MB-231). Second, the expression of signaling-related genes was examined in publicly available breast cancer cohorts with respect to correlation, tumor versus normal tissue, and overall survival. The two steps are complementary rather than confirmatory, since the array measures protein phosphorylation and the cohort analyses measure transcript abundance; this distinction is addressed explicitly in the Discussion.

## 2. Results

### 2.1. Phospho-Kinase Array Profiling of ACh and PRL Responses

To obtain a comprehensive view of the signaling networks modulated by ACh and PRL, the Proteome Profiler™ Human Phospho-Kinase Array Kit (R&D Systems, Minneapolis, MN, USA, Cat. No. ARY003C) was employed. Full phosphorylation profiles for all targets are shown in Figure 1 for each cell line.

The utilization of the Proteome Profiler^TM^ Human Phospho-Kinase Array Kit, which simultaneously detects the relative phosphorylation of 37 kinase phosphorylation sites and 2 total proteins on duplicate nitrocellulose membranes [24], allowed for a comprehensive snapshot of the signaling networks active in response to ACh and PRL across three distinct breast cancer subtypes: the luminal/estrogen receptor-positive lines (MCF7, T47D) and a triple-negative line (MDA-MB-231). The data demonstrated that both ACh and PRL individually modulated the phosphorylation status of numerous kinases; however, the combination treatment (ACh+PRL) induced unique and cell-type-specific signaling signatures, suggesting complex crosstalk between these factors.

The MCF7 and T47D cell lines, characterized by a dependency on estrogen signaling, showed pronounced modulation in pathways typically associated with growth and proliferation, such as the PI3K/AKT pathway (p-AKT) and aspects of the MAPK cascade. Both single treatments tended to activate these survival pathways, suggesting that both ACh and PRL might promote a proliferative phenotype dependent on estrogen signaling mechanisms. On the other hand, the MDA-MB-231 cell line exhibited basal activation in different profiles compared to the ER+ lines. Its response to ACh and PRL suggested that a potentially more diverse or less uniformly estrogen-dependent signaling network was engaged. The combination treatment might be particularly critical here, as these highly aggressive lines often rely on multiple compensatory pathways for survival.

Concerning the signal transduction pathways affected by single treatments (ACh or PRL), the activation of growth and survival pathways indicated that across all cell lines, treatments often resulted in the phosphorylation (activation) of kinases that mediated cell survival and metabolism. For instance, several targets within the PI3K/AKT pathway appeared activated following single treatment with either ACh or PRL compared to untreated controls. This suggested a common pro-survival signal induced by both factors, potentially independent of estrogen receptor status. The modulation of pERK (part of the MAPK cascade) was evident in MCF7 and T47D cells, suggesting that these factors stimulated pathways related to cell cycle progression.

The most striking biological observation came from analyzing the combination treatment (ACh + PRL) group. In several instances, the pattern observed in the ACh + PRL group was quantitatively and qualitatively distinct from the summation of single treatments (ACh vs. Untreated or PRL vs. Untreated). This deviation strongly suggested that ACh and PRL modulated signaling through non-redundant mechanisms, activating completely separate kinase targets or modifying existing pathways in a synergistic manner (i.e., non-redundant signaling). On the other hand, there was a potential for over-activation/inhibition. Depending on the specific target (e.g., certain p38 sites), the combination might lead to an over-activation signal (hyperphosphorylation) compared to single doses, potentially pushing the cell towards uncontrolled signaling. Conversely, for other targets, the combination might stabilize or inhibit a pathway, which could represent an antiproliferative mechanism.

Statistically, the observed phosphorylation changes were generally significant relative to the untreated control across multiple kinases in all tested conditions, confirming that ACh and PRL were potent modulators of cellular signaling. The heterogeneity of modulation (some sites activated, some inhibited) mandated a detailed investigation into specific targets. The differential effects between cell lines confirmed that these hormonal/neuromodulatory signals did not act uniformly; the underlying molecular context dictated the biological outcome. In summary, ACh and PRL modulated diverse kinase networks in breast cancer cells via complex crosstalk. Their combined action (ACh + PRL) suggested a synergistic signaling mechanism capable of altering fundamental growth and survival pathways differently than either factor alone, potentially providing novel therapeutic targets for combination therapy development.

From the global array, a targeted panel was selected to facilitate the interpretation and visualization of treatment-associated signaling responses. The selected panel was designed to capture convergent signaling responses to ACh, PRL, and their combination across receptor-proximal, cytoplasmic kinase, and transcriptional pathways. AKT1/2/3, p70S6K/p70S6K1, and RSK1/2/3 represented survival, growth, translational, and downstream effector signaling, whereas EGFR and ERK1/2 captured receptor-associated MAPK activity. PLC-γ1, Src/SRC, and Yes/YES1 provided information about receptor-linked and Src-family signaling, while p53, STAT3, and c-JUN reflected complementary stress-response and transcriptional outputs. Overall, the panel was used to identify treatment-associated phosphorylation patterns and potential pathway convergence rather than to reconstruct complete ACh- or PRL-signaling pathways [24,25,26,27]. 

### 2.2. ACh and PRL Regulate AKT, p70S6K and RSK1/2/3 in a Subtype-Dependent Manner

To assess the effects of ACh and PRL on transcriptional signaling, phosphorylation of AKT, p70S6K, and RSK was quantified in MCF7, T47D, and MDA-MB-231 breast cancer cell lines following 4 h treatment with ACh, PRL, and ACh + PRL (Figure 2).

As shown in Figure 2A, AKT1/2/3 phosphorylation at Ser473 was significantly (*p* < 0.05) increased by ACh alone and also increased by the combination of ACh + PRL (*p* < 0.001) in the MCF7 cell line, while PRL alone was not significant. In the T47D cell line, only the ACh + PRL combination significantly (*p* < 0.05) decreased p-AKT-S473. In the MDA-MB-231 cell line, neither ACh nor PRL alone reached statistical significance, despite showing trends toward a decrease and an increase, respectively. The ACh + PRL combination significantly increased phosphorylation of AKT-T308 in MCF7 (*p* < 0.001) and MDA-MB-231 (*p* < 0.001); PRL alone significantly (*p* < 0.05) increased it in MDA-MB-231; whereas a significant decrease (*p* < 0.01) was observed with ACh alone in the T47D cell line, no other significant changes were detected in this cell line (Figure 2B). p70S6K phosphorylation (T421/S424) was markedly increased by ACh alone in MCF7 (approximately 9-fold; *p* < 0.001), and by ACh + PRL in T47D (*p* < 0.001); no significant changes were detected in MDA-MB-231 (Figure 2C). RSK1/2/3 phosphorylation (S380/S386/S377) showed no significant changes in the MCF7 cell line, whereas PRL alone significantly (*p* < 0.05) decreased RSK phosphorylation in the T47D cell line. In contrast, ACh alone (*p* < 0.05) and ACh + PRL (*p* < 0.001) significantly increased p-RSK1/2/3 in MDA-MB-231 (Figure 2D). Taken together, the largest single-agent response of this group was the ACh-induced increase in p70S6K phosphorylation in MCF7, which was not reproduced in T47D or MDA-MB-231. This indicates that the same stimulus engages the mTOR/S6K branch only in a permissive cellular context rather than uniformly across breast cancer subtypes.

**Figure 2:**
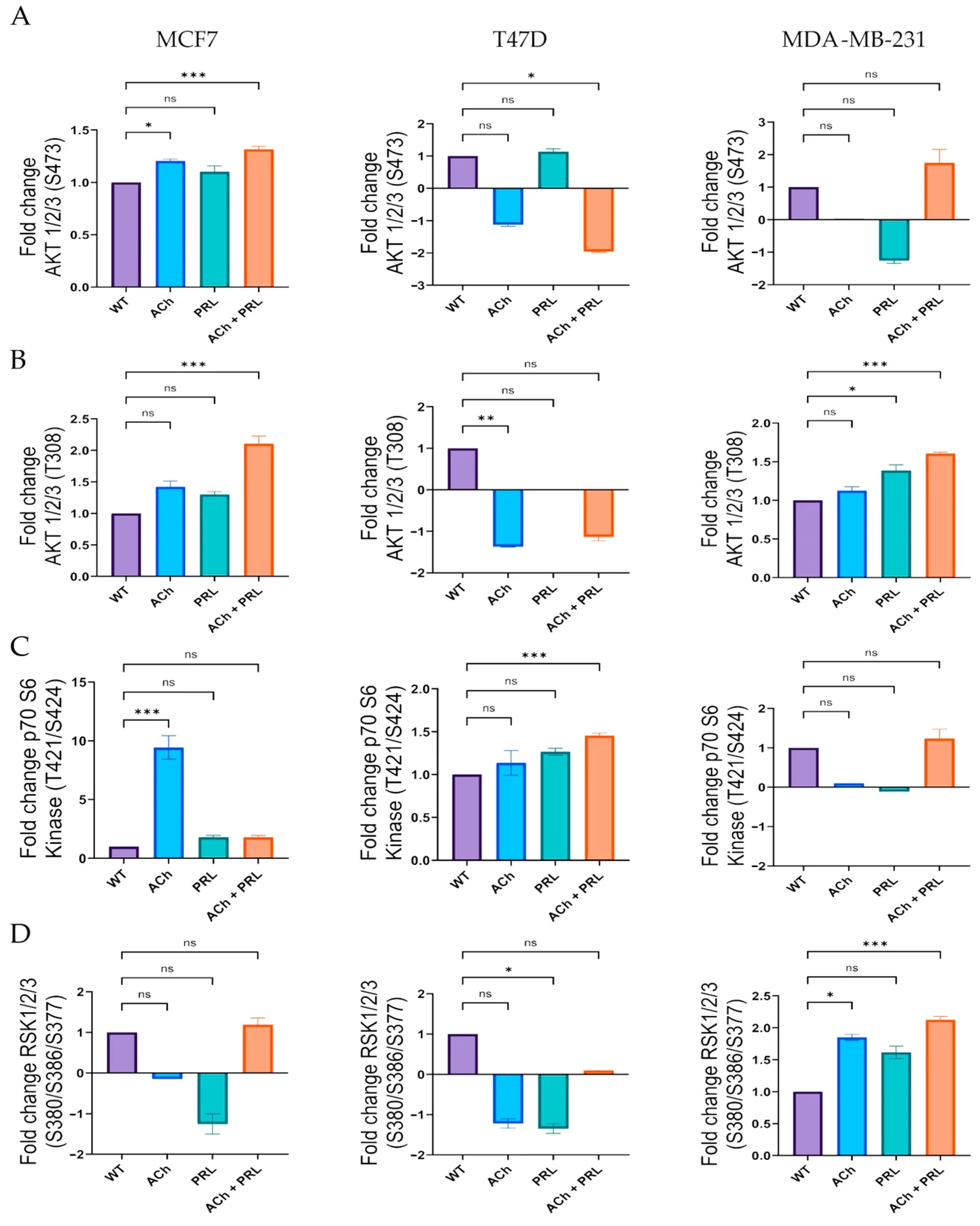
Phosphorylation of (**A**) AKT1/2/3 (S473), (**B**) AKT1/2/3 (T308), (**C**) p70S6 Kinase (T421/S424) and (**D**) RSK1/2/3 (S380/S386/S377) in MCF7, T47D, and MDA-MB-231 cell lines after 4 h treatment with ACh, PRL, or combination of ACh and PRL, determined by phosphoproteomic array. Data are expressed as mean ± SEM of three independent experiments. * *p* < 0.05, ** *p* < 0.01, *** *p* < 0.001 vs. control; ns = not significant.

### 2.3. Expression of PI3K/AKT and S6-Kinase Genes in Breast Cancer Cohorts

To evaluate the transcriptional associations and prognostic relevance of selected genes related to AKT–S6 kinase signaling in breast cancer, publicly available breast cancer transcriptomic datasets were analyzed. Specifically, the co-expression patterns, differential expression in tumor versus normal tissues, and overall survival associations of *ACHE* and *PRLR* were assessed with key downstream effectors, namely the AKT isoforms (*AKT1*, *AKT2*, *AKT3*) and the ribosomal protein S6 kinase family (*RPS6KB1*, *RPS6KA1*, *RPS6KA3*, *RPS6KA2*), as illustrated in Figure 3.

The correlation analysis presented in Figure 3A identified subtype-dependent associations between *ACHE* expression and selected genes encoding AKT isoforms, p70S6K, and RSK-family proteins in breast cancer. In the overall breast cancer cohort, *ACHE* expression showed no significant correlation with *AKT1* or *AKT2*. However, significant positive correlations were observed with *AKT3* (ρ = 0.150, *p* = 6.44 × 10^−7^) and several downstream ribosomal protein S6 kinases (RPS6Ks). Specifically, *ACHE* was positively correlated with *RPS6KA1* (ρ = 0.145, *p* = 1.49 × 10^−6^), *RPS6KA2* (ρ = 0.158, *p* = 1.75 × 10^−7^), and most strongly with *RPS6KA3* (ρ = 0.302, *p* = 2.31 × 10^−24^). Conversely, a significant negative correlation was observed with *RPS6KB1* (ρ = −0.076, *p* = 0.012).

In the Basal-like breast cancer subtype, *ACHE* expression did not correlate significantly with *AKT1*, *AKT2*, *RPS6KB1*, or *RPS6KA1*. Significant positive correlations were found with *AKT3* (ρ = 0.177, *p* = 0.015), *RPS6KA3* (ρ = 0.146, *p* = 0.045), and *RPS6KA2* (ρ = 0.197, *p* = 0.007). The HER2-enriched subtype exhibited a restricted correlation profile. *ACHE* expression showed no significant correlation with *AKT1*, *AKT2*, *AKT3*, *RPS6KB1*, *RPS6KA1*, or *RPS6KA2*. The only statistically significant positive correlation in this subtype was with *RPS6KA3* (ρ = 0.220, *p* = 0.047).

The Luminal A subtype displayed a broad correlation profile. While *ACHE* did not correlate with *AKT1*, it showed a weak but significant positive correlation with *AKT2* (ρ = 0.084, *p* = 0.046). Stronger significant positive correlations were observed with *AKT3* (ρ = 0.152, *p* = 3.17 × 10^−4^), *RPS6KA3* (ρ = 0.240, *p* = 8.47 × 10^−9^), and *RPS6KA2* (ρ = 0.150, *p* = 3.73 × 10^−4^). The correlations with *RPS6KB1* and *RPS6KA1* were non-significant.

In the Luminal B subtype, *ACHE* expression did not correlate significantly with *AKT1*, *AKT2*, *AKT3*, or *RPS6KB1*. Significant positive correlations were restricted to the RPS6KA family, *RPS6KA1* (ρ = 0.141, *p* = 0.039), *RPS6KA3* (ρ = 0.323, *p* = 1.27 × 10^−6^), and *RPS6KA2* (ρ = 0.180, *p* = 0.008).

The correlation analysis for *PRLR* showed positive associations with a broader set of AKT–S6 kinase-related genes, in comparison with *ACHE* analysis, across the overall breast cancer cohort and molecular subtypes, including *AKT1*, *AKT2*, *AKT3*, *RPS6KB1*, *RPS6KA3*, and *RPS6KA2*. In the overall breast cancer cohort, *PRLR* expression exhibited robust and highly significant positive correlations with all three upstream AKT isoforms: *AKT1* (ρ = 0.256, *p* = 9.49 × 10^−1^), *AKT2* (ρ = 0.280, *p* = 5.65 × 10^−21^), and *AKT3* (ρ = 0.185, *p* = 8.83 × 10^−1^). Among the downstream effectors, *PRLR* showed an exceptionally strong positive correlation with *RPS6KB1* (ρ = 0.510, *p* = 6.43 × 10^−73^). Additionally, significant positive correlations were observed with *RPS6KA3* (ρ = 0.079, *p* = 0.009) and *RPS6KA2* (ρ = 0.272, *p* = 6.44 × 10^−20^). No significant correlation was found with *RPS6KA1*.

In the Basal-like subtype, *PRLR* expression showed significant positive correlations with *AKT1* (ρ = 0.220, *p* = 0.002) and *AKT2* (ρ = 0.193, *p* = 0.008), but not with *AKT3*. Downstream, the strongest correlation was observed with *RPS6KA3* (ρ = 0.421, *p* = 1.74 × 10^−9^), alongside a highly significant positive correlation with *RPS6KB1* (ρ = 0.405, *p* = 8.31 × 10^9^). A significant positive correlation was also maintained with *RPS6KA2* (ρ = 0.146, *p* = 0.046). *RPS6KA1* did not show a significant correlation. The HER2-enriched subtype displayed a distinct correlation profile, with *PRLR* strongly correlating with *AKT3* (ρ = 0.393, *p* = 0.00026), while correlations with *AKT1* and *AKT2* were non-significant. Downstream, *PRLR* exhibited a very strong positive correlation with *RPS6KB1* (ρ = 0.481, *p* = 4.71 × 10^−6^), as well as significant positive correlations with *RPS6KA3* (ρ = 0.237, *p* = 0.032) and *RPS6KA2* (ρ = 0.316, *p* = 0.004).

The Luminal A subtype exhibited strong positive correlations between *PRLR* and *AKT2* (ρ = 0.250, *p* = 2.00 × 10^−9^) and *AKT3* (ρ = 0.345, *p* = 4.26 × 10^−17^). The correlation with *AKT1* was borderline non-significant (ρ = 0.076, *p* = 0.071). Downstream, *PRLR* showed a positive correlation with *RPS6KB1* (ρ = 0.483, *p* = 4.64 × 10^−34^), with *RPS6KA3* (ρ = 0.267, *p* = 1.39 × 10^−10^) and *RPS6KA2* (ρ = 0.214, *p* = 3.25 × 10^−7^). In the Luminal B subtype, *PRLR* expression was positively correlated with all three AKT isoforms: *AKT1* (ρ = 0.141, *p* = 0.038), *AKT2* (ρ = 0.233, *p* = 0.00057), and *AKT3* (ρ = 0.207, *p* = 0.002). Downstream, significant positive correlations were maintained with *RPS6KB1* (ρ = 0.368, *p* = 2.58 × 10^−^), *RPS6KA3* (ρ = 0.236, *p* = 0.00048), and *RPS6KA2* (ρ = 0.231, *p* = 0.00061).

To characterize tumor-versus-normal expression patterns in genes related to AKT–S6 kinase signaling, the mRNA levels of *AKT1*, *AKT2*, *AKT3*, *RPS6KB1*, and selected *RPS6KA* family genes were compared between breast tumor tissues and matched normal adjacent tissues. As shown in Figure 3C, distinct expression patterns emerged among the pathway components. The expression of *AKT1* and *RPS6KA1* was significantly higher in tumor tissues compared to normal tissues (*p* < 0.001). In contrast, *AKT3*, *RPS6KA3*, and *RPS6KA2* exhibited significantly higher expression in normal tissues compared to tumor tissues (*p* < 0.001). *AKT2* and *RPS6KB1* expression showed no statistically significant difference between tumor and normal tissues.

To evaluate whether expression of selected AKT- and S6 kinase-related genes was associated with overall survival, Kaplan–Meier analyses were performed in the breast cancer cohort. For each gene, patients were divided into low- and high-expression groups using the median transcript level as the cutoff. Among the AKT isoforms, high expression of *AKT3* was significantly associated with improved overall survival (HR = 0.71, 95% CI: 0.58–0.85, log-rank *p* = 0.00028). In contrast, the expression levels of *AKT1* and *AKT2* did not significantly impact patient survival. Within the RPS6K family, high expression of *RPS6KB1* was significantly associated with improved overall survival (HR = 0.69, 95% CI: 0.53–0.90, log-rank *p* = 0.0066). Similarly, high expression of *RPS6KA1* was significantly associated with a favorable prognosis (HR = 0.79, 95% CI: 0.66–0.96, log-rank *p* = 0.015). High expression of *RPS6KA2* (HR = 0.84, 95% CI: 0.69–1.01, log-rank *p* = 0.064) and *RPS6KA3* (HR = 0.85, 95% CI: 0.65–1.11, log-rank *p* = 0.24) showed trends toward improved survival, though these associations did not reach statistical significance.

### 2.4. ACh and PRL Modulate EGFR and ERK1/2 Phosphorylation

To determine whether ACh and PRL regulate the EGFR/MAPK signaling pathway, phosphorylation levels of EGFR and its downstream effector ERK1/2 were evaluated in MCF7, T47D, and MDA-MB-231 breast cancer cell lines following 4 h treatment with ACh, PRL, or their combination (Figure 4).

EGFR phosphorylation (Y1086) was significantly decreased by ACh (*p* < 0.05) and PRL (*p* < 0.01) in the MCF7 cell line; no significant changes were detected in the T47D cell line; in MDA-MB-231, PRL significantly (*p* < 0.01) increased pEGFR, while ACh and ACh + PRL were not significant (Figure 4A).

ERK1/2 phosphorylation (T202/Y204, T185/Y187) was significantly decreased by ACh (*p* < 0.05) and PRL (*p* < 0.001) in MCF7, and by ACh + PRL (*p* < 0.001) in T47D; in MDA-MB-231, ACh alone significantly (*p* < 0.01) increased pERK1/2, while PRL and ACh + PRL were not significant (Figure 4B). Both markers therefore behaved in opposite directions in the luminal and the triple-negative context: ACh reduced EGFR and ERK1/2 phosphorylation in MCF7 but increased ERK1/2 phosphorylation in MDA-MB-231.

**Figure 4:**
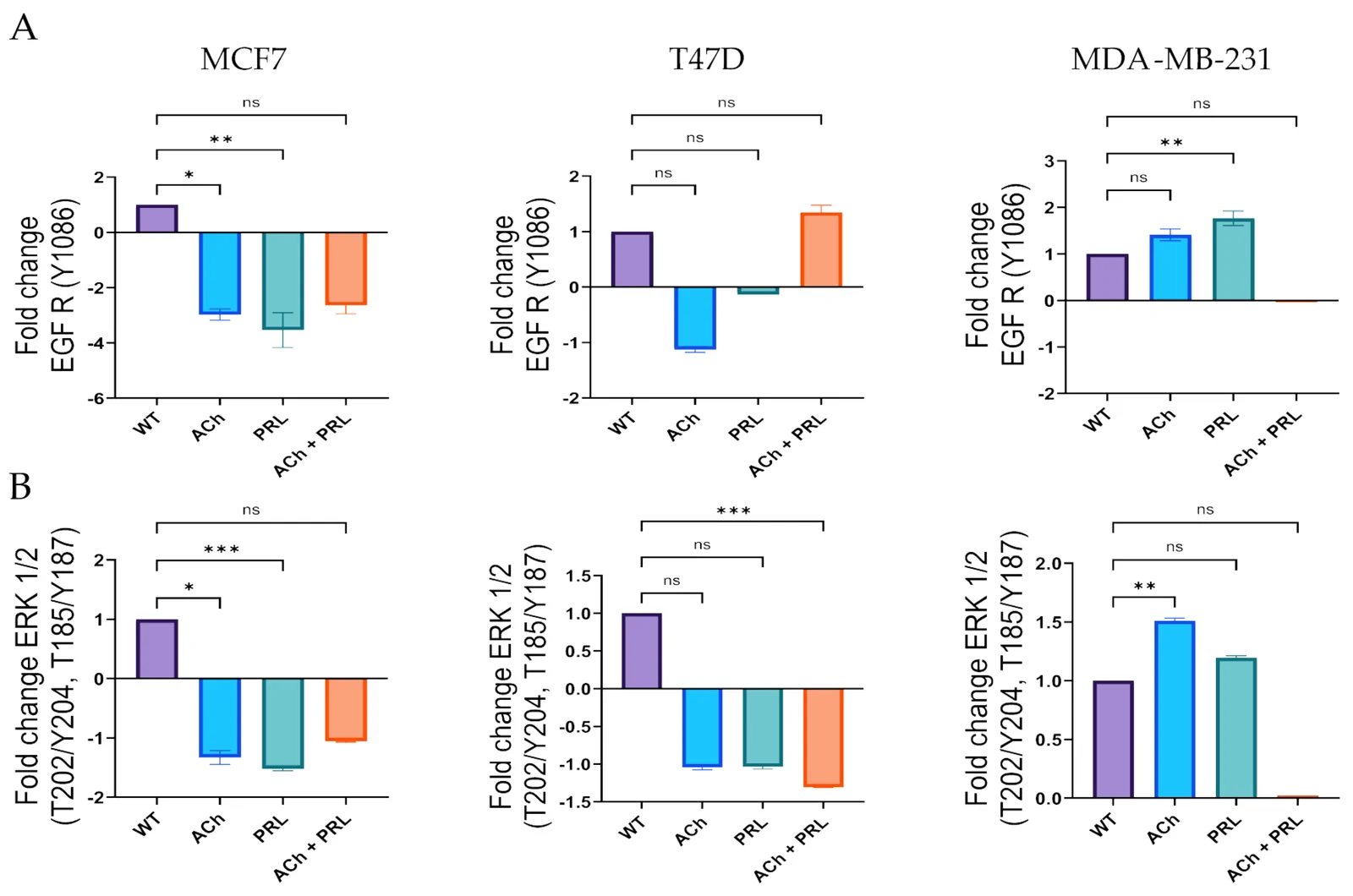
Quantification of (**A**) EGF R (Y1086) and (**B**) ERK1/2 (T202/Y204, T185/Y187) phosphorylation in MCF7, T47D, and MDA-MB-231 cell lines after 4 h treatment with ACh, PRL, or ACh + PRL. Data are expressed as the mean ± SEM of three independent experiments. * *p* < 0.05, ** *p* < 0.01, *** *p* < 0.001 vs. control. ns: non-significant.

### 2.5. Expression of PRLR and ACHE, EGFR and MAPK Pathway Genes in Breast Cancer Cohorts

To characterize subtype-specific transcriptomic and survival associations of genes responsive to ACh- and PRL-associated treatments in vitro, publicly available breast cancer transcriptomic data were analyzed. Specifically, the associations of *ACHE* and *PRLR* expression with selected EGFR–ERK-related genes, *EGFR*, *MAPK3* (ERK1), and *MAPK1* (ERK2), were examined, together with their expression differences between tumor and normal tissues and their relationships with overall survival, as shown in Figure 5.

The correlation analysis presented in Figure 5A identifies associations between *ACHE* expression and selected EGFR–ERK-related genes, with these associations differing across molecular subtypes of breast cancer. In the overall breast cancer cohort, *ACHE* expression exhibited a strong and highly significant positive correlation with *EGFR* (ρ = 0.346, *p* = 5.88 × 10^−32^). In contrast, *ACHE* showed no significant correlation with the downstream MAPK cascade effectors, *MAPK3* (ERK1) or *MAPK1* (ERK2). In the Basal-like subtype, *ACHE* expression was uncoupled from the EGFR/MAPK pathway. There were no statistically significant correlations between *ACHE* and *EGFR*, *MAPK3*, or *MAPK1*.

The HER2-enriched subtype displayed a distinct correlation profile. *ACHE* expression showed significant positive correlations with both the receptor *EGFR* (ρ = 0.291, *p* = 0.008) and the downstream kinase *MAPK3* (ρ = 0.248, *p* = 0.025). The correlation with *MAPK1* showed a trend toward a negative association but did not reach statistical significance (ρ = −0.196, *p* = 0.078). In the Luminal A subtype, *ACHE* expression demonstrated a robust and highly significant positive correlation with *EGFR* (ρ = 0.294, *p* = 1.20 × 10^−12^). Similar to the overall cohort, *ACHE* did not significantly correlate with *MAPK3* or *MAPK1.* In the Luminal B subtype, *ACHE* expression was significantly and positively correlated with *EGFR* (ρ = 0.207, *p* = 0.002). No significant correlations were found with *MAPK3* or *MAPK1*.

The correlation analysis for *PRLR* identifies associations with selected EGFR–ERK-related genes, with the strength and direction of these associations differing between receptor-level and downstream effector-level genes (Figure 5B). In the overall breast cancer cohort, *PRLR* expression exhibited a significant negative correlation with the receptor *EGFR* (*ρ* = −0.148, *p* = 9.65 × 10^−7^). In contrast, *PRLR* showed robust and highly significant positive correlations with the downstream MAPK cascade effectors, specifically *MAPK3* (ERK1; *ρ* = 0.272, *p* = 8.17 × 10^−20^) and *MAPK1* (ERK2; *ρ* = 0.461, *p* = 3.44 × 10^−5^). In the Basal-like subtype, the relationship between *PRLR* and *EGFR* shifted to a significant positive correlation (*ρ* = 0.237, *p* = 0.001). *PRLR* also maintained a very strong positive correlation with *MAPK1* (*ρ* = 0.476, *p* = 4.87 × 10^−12^). However, no significant correlation was observed with *MAPK3*.

The HER2-enriched subtype displayed the most restricted correlation profile. *PRLR* expression showed a highly significant and strong positive correlation exclusively with *MAPK1* (*ρ* = 0.637, *p* = 1.25 × 10^−10^). No significant correlations were found with *EGFR* or *MAPK3*. In the Luminal A subtype, *PRLR* expression was significantly and positively correlated with both *EGFR* (*ρ* = 0.155, *p* = 2.31 × 10^−4^) and *MAPK1* (*ρ* = 0.548, *p* = 4.00 × 10^−4^). Similar to the Basal subtype, *PRLR* did not significantly correlate with *MAPK3*. In the Luminal B subtype, *PRLR* expression demonstrated significant positive correlations with *EGFR* (*ρ* = 0.170, *p* = 0.012) and *MAPK1* (*ρ* = 0.482, *p* = 5.48 × 10^−14^). No significant correlation was observed with *MAPK3*.

To evaluate differences in the expression of selected components related to EGFR–ERK signaling in breast cancer, mRNA expression levels of *EGFR*, *MAPK3* (ERK1), and *MAPK1* (ERK2) were compared between breast tumor tissues and matched normal adjacent tissues. As shown in Figure 5C, *EGFR* expression was significantly higher in normal breast tissues compared to tumor tissues (*p* < 0.001). Similarly, *MAPK1* expression was significantly elevated in normal tissues relative to tumor tissues (*p* < 0.01). In contrast, *MAPK3* expression showed no statistically significant difference between tumor and normal breast tissues.

The association between *EGFR*, *MAPK3* (ERK1), and *MAPK1* (ERK2) expression and breast cancer patient outcomes was evaluated using Kaplan–Meier survival analyses. Patients were stratified into low- and high-expression groups based on the median transcript level of each gene. For *EGFR*, no significant association between expression levels and overall survival was observed. In contrast, a significant association between *MAPK3* expression and overall survival was identified; a higher survival probability was exhibited by the high-expression group, indicating a favorable prognosis (HR = 0.76, 95% CI: 0.63–1.92, log-rank *p* = 0.0042). For *MAPK1*, a trend toward poorer overall survival was indicated by high expression levels, although this association was borderline significant (HR = 1.31, 95% CI: 1.00–1.71, log-rank *p* = 0.05).

### 2.6. ACh and PRL Modulate p53, PLCγ1, SRC and YES1 Phosphorylation

To further characterize the downstream signaling effects of ACh and PRL, phosphorylation of proteins involved in cell stress responses, signal transduction, and tyrosine kinase activity, including p53, PLCγ1, Src, and YES1, was evaluated in MCF7, T47D, and MDA-MB-231 breast cancer cell lines following 4 h treatment with ACh, PRL, or their combination, as seen in Figure 6.

**Figure 6:**
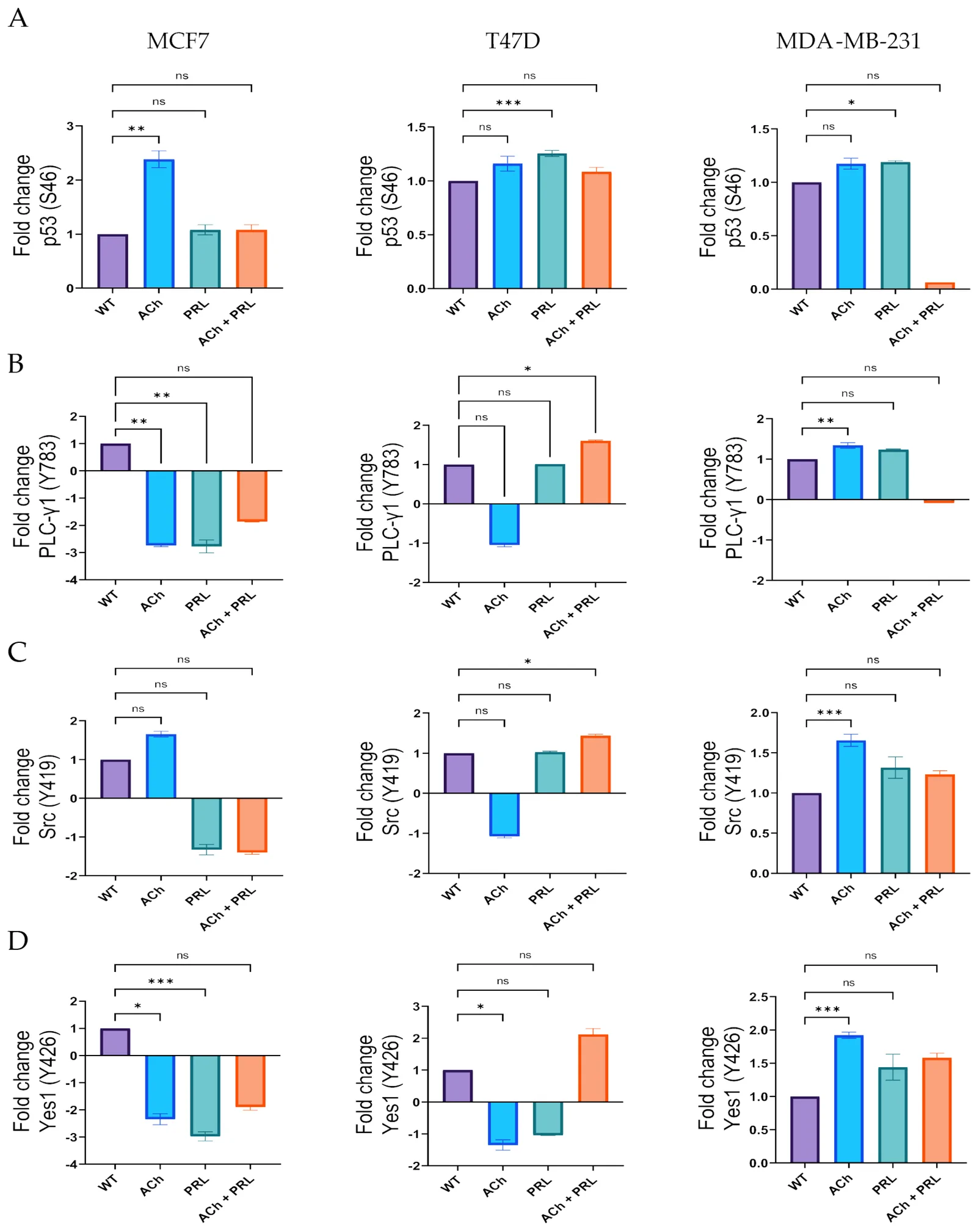
Quantification of (**A**) p53 (S46), (**B**) PLCγ1 (Y783), (**C**) SRC (Y419), and (**D**) YES1 (Y426) phosphorylation in MCF7, T47D, and MDA-MB-231 cell lines after 4 h treatment with ACh, PRL, or ACh + PRL. Data are expressed as mean ± SEM of three independent experiments. * *p* < 0.05, ** *p* < 0.01, *** *p* < 0.001 vs. control.

As shown in Figure 6A, p53 phosphorylation (S46) was significantly increased by ACh in MCF7 (*p* < 0.01); by PRL alone in T47D (*p* < 0.001); and by PRL alone in MDA-MB-231 (*p* < 0.05); no other treatments produced statistically significant changes in the cell lines.

PLCγ1 phosphorylation (Y783) was significantly decreased by both ACh (*p* < 0.01) and PRL (*p* < 0.01) individually in the MCF7 cell line; in T47D, the ACh + PRL combination significantly (*p* < 0.05) increased pPLCγ1; ACh alone significantly (*p* < 0.01) increased pPLCγ1 in the MDA-MB-231 cell line (Figure 6B).

SRC phosphorylation (Y419) showed no significant changes in the MCF7 cell line. ACh + PRL combined significantly (*p* < 0.05) increased p-SRC in T47D; ACh alone significantly (*p* < 0.001) increased p-SRC in MDA-MB-231 (Figure 6C).

YES1 phosphorylation (Y426) was significantly decreased by ACh (*p* < 0.05) and PRL (*p* < 0.001) in the MCF7 cell line. ACh alone significantly (*p* < 0.05) decreased p-YES1 in T47D, and ACh alone significantly (*p* < 0.001) increased p-YES1 in MDA-MB-231 (Figure 6D).

### 2.7. Expression of TP53, PLCG1, SRC and YES1 in Breast Cancer Cohorts

Figure 7 presents the results of the analyses involving selected genes related to TP53/SRC signaling. First, correlations between *ACHE* and *PRLR* expression and the selected genes *TP53*, phospholipase C gamma 1 (*PLCG1*), proto-oncogene tyrosine-protein kinase (*SRC*), and YES proto-oncogene 1 (*YES1*) were assessed. Next, the expression of these four genes was compared between breast tumor and normal adjacent tissues. Finally, their associations with overall survival were evaluated.

The correlation analysis presented in Figure 7A shows subtype-specific differences in the associations between *ACHE* expression and the selected genes *TP53*, *PLCG1*, *SRC*, and *YES1*. In the overall breast cancer cohort, *ACHE* expression exhibited significant positive correlations with *PLCG1* (ρ = 0.202, *p* = 1.74 × 10^−11^) and *SRC* (ρ = 0.137, *p* = 6.31 × 10^−6^), whereas no statistically significant correlations were observed between *ACHE* and *TP53* or *YES1*.

In the Basal-like subtype, the correlation profile shifted distinctly. *ACHE* expression showed a significant positive correlation with *TP53* (ρ = 0.210, *p* = 0.0038) and a significant negative correlation with *YES1* (ρ = −0.173, *p* = 0.018). The associations with *PLCG1* and *SRC* were not statistically significant. The HER2-enriched subtype displayed a complete decoupling of *ACHE* from the evaluated TP53/SRC network. No significant correlations were found between *ACHE* expression and *TP53*, *PLCG1*, *SRC*, or *YES1*. In the Luminal A subtype, *ACHE* expression demonstrated a robust positive correlation with *PLCG1* (ρ = 0.263, *p* = 2.70 × 10^−10^) and a weaker, yet significant, positive correlation with *SRC* (ρ = 0.089, *p* = 0.034). Additionally, a significant negative correlation was observed with *YES1* (ρ = −0.114, *p* = 0.007). No significant correlation was found with *TP53*. In the Luminal B subtype, *ACHE* expression was significantly and positively correlated with both *PLCG1* (ρ = 0.194, *p* = 0.0043) and *SRC* (ρ = 0.235, *p* = 0.0005). Similar to the overall cohort, no significant correlations were detected with *TP53* or *YES1*.

The correlation analysis for *PRLR* shows a different pattern of associations with the selected TP53/SRC-related genes compared with *ACHE*, with positive correlations observed for several of the analyzed molecular subtypes. In the overall breast cancer cohort, *PRLR* expression exhibited significant positive correlations with *TP53* (ρ = 0.135, *p* = 7.69 × 10^−6^), *PLCG1* (ρ = 0.150, *p* = 6.76 × 10^−7^), and *YES1* (ρ = 0.161, *p* = 9.64 × 10^−8^). No statistically significant correlation was observed between *PRLR* and *SRC*.

In the Basal-like subtype, *PRLR* expression showed a remarkably strong and highly significant positive correlation with *YES1* (ρ = 0.543, *p* = 8.34 × 10^−16^), alongside a significant positive correlation with *PLCG1* (ρ = 0.231, *p* = 0.0014). Conversely, no significant correlations were detected with *TP53* or *SRC*. The HER2-enriched subtype displayed significant positive correlations between *PRLR* and both *PLCG1* (ρ = 0.250, *p* = 0.024) and *YES1* (ρ = 0.303, *p* = 0.0056). Additionally, borderline significant trends were observed for *TP53* (ρ = 0.216, *p* = 0.051) and *SRC* (ρ = 0.209, *p* = 0.060) but did not reach statistical significance. In the Luminal A subtype, *PRLR* expression demonstrated a broad and robust integration with the entire evaluated network. Significant positive correlations were identified with *TP53* (ρ = 0.161, *p* = 1.35 × 10^−4^), *PLCG1* (ρ = 0.211, *p* = 4.62 × 10^−7^), *SRC* (ρ = 0.115, *p* = 0.0063), and *YES1* (ρ = 0.512, *p* = 8.10 × 10^−39^). In the Luminal B subtype, *PRLR* expression was significantly and positively correlated with *PLCG1* (ρ = 0.174, *p* = 0.010) and *YES1* (ρ = 0.232, *p* = 5.99 × 10^−4^). A borderline trend toward significance was noted for *TP53* (ρ = 0.127, *p* = 0.063) but did not reach statistical significance, while no significant correlation was found with *SRC*.

The mRNA expression levels of *TP53*, *PLCG1*, *SRC*, and *YES1* were compared between breast tumor and matched normal adjacent tissues to assess differences in expression of these selected genes. As shown in Figure 7C, *SRC* expression was significantly higher in tumor tissues compared to normal tissues (*p* < 0.001). Conversely, *YES1* expression was significantly higher in normal tissues compared to tumor tissues (*p* < 0.05). In contrast, no statistically significant differences in expression levels between tumor and normal tissues were observed for *TP53* or *PLCG1*.

The associations between *TP53*, *PLCG1*, *SRC*, and *YES1* expression and overall survival in breast cancer patients were evaluated using Kaplan–Meier survival analyses. Patients were stratified into low and high expression groups based on median transcript levels. High expression of *YES1* was significantly associated with poorer overall survival (HR = 1.24, 95% CI: 1.02–1.49, log-rank *p* = 0.028). In contrast, the expression levels of *TP53*, *PLCG1*, and *SRC* did not show statistically significant associations with patient overall survival.

### 2.8. ACh and PRL Modulate STAT3 and c-Jun Phosphorylation

To assess the effects of ACh and PRL on transcriptional signaling, phosphorylation of STAT3 and c-JUN was quantified in MCF7, T47D, and MDA-MB-231 breast cancer cell lines after 4 h of stimulation with ACh, PRL, or combined ACh + PRL (Figure 8).

STAT3 (S727) phosphorylation was significantly increased by the combination of ACh + PRL (*p* < 0.05) in MCF7; it was significantly (*p* < 0.01) downregulated by ACh in the T47D cell line, and by the ACh + PRL combination (*p* < 0.001) in MDA-MB-231 (Figure 8A).

c-Jun phosphorylation (S63) was not significantly changed by any treatment in MCF7. ACh + PRL significantly (*p* < 0.01) decreased p-c-Jun in T47D, with no significant changes for ACh or PRL alone; the ACh + PRL combination significantly (*p* < 0.001) increased p-c-Jun in MDA-MB-231, while individual treatments were not significant (Figure 8B).

**Figure 8:**
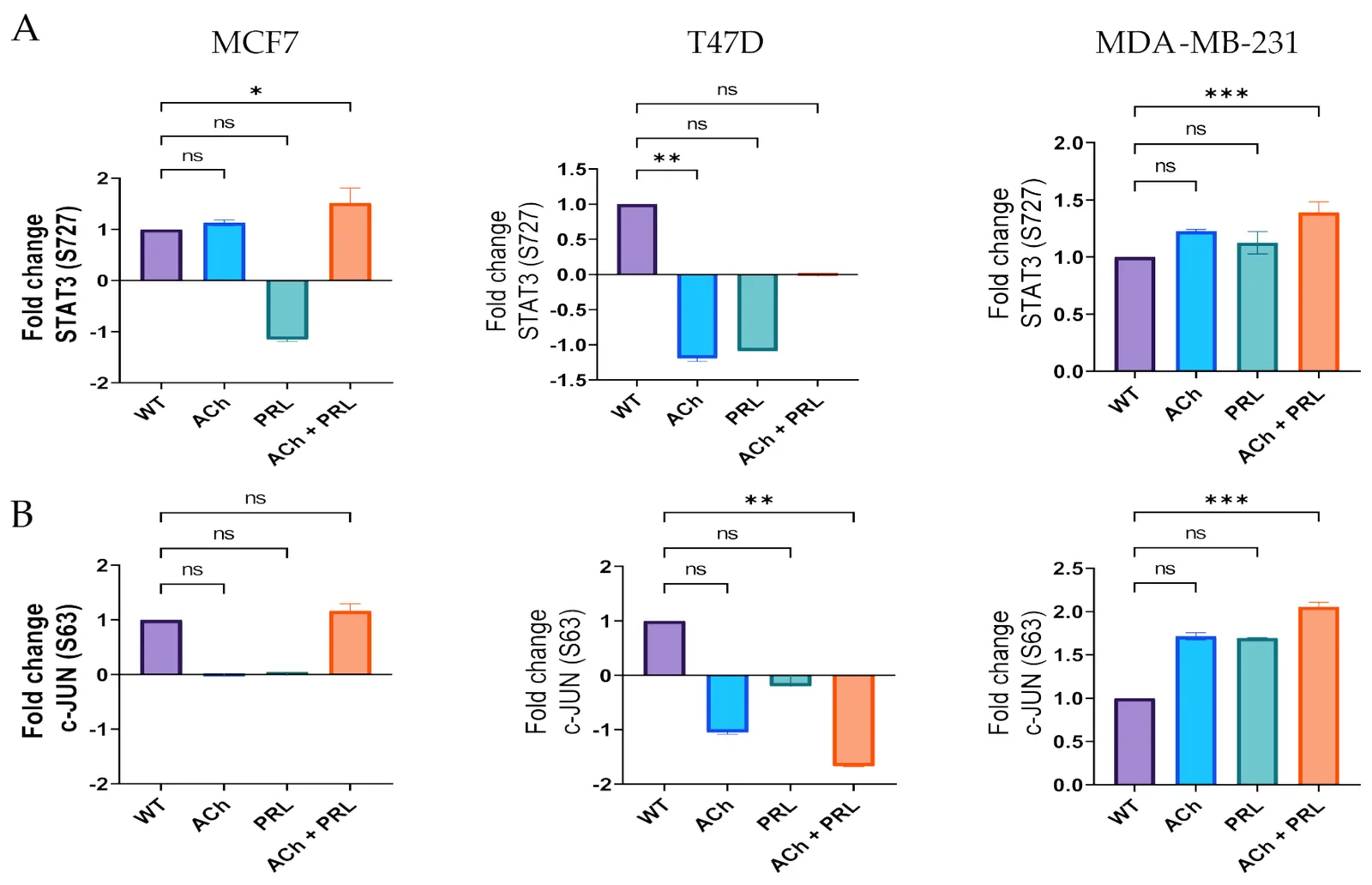
Quantification of (**A**) STAT3 (S727) and (**B**) c-JUN (S63) phosphorylation in MCF7, T47D, and MDA-MB-231 cell lines after 4 h treatment with ACh, PRL, or ACh + PRL. Data are expressed as mean ± SEM of three independent experiments. * *p* < 0.05, ** *p* < 0.01, *** *p* < 0.001 vs. control.

### 2.9. Expression of STAT3 and JUN in Breast Cancer Cohorts

To assess the associations between the non-canonical receptors *ACHE* and *PRLR* and selected genes related to STAT3/JUN signaling, correlations between *ACHE* and *PRLR* expression and signal transducer and activator of transcription 3 (*STAT3*) and Jun proto-oncogene (*JUN*) were examined. In addition, the expression of *STAT3* and *JUN* was compared between breast tumor and matched normal adjacent tissues, and their associations with overall survival were evaluated, as illustrated in Figure 9.

The correlation analysis presented in Figure 9A shows subtype-specific differences in the associations between *ACHE* expression and *STAT3* and *JUN* expression. In the overall breast cancer cohort, *ACHE* expression exhibited a weak but statistically significant negative correlation with *STAT3* (ρ = −0.061, *p* = 0.045). Conversely, *ACHE* demonstrated a highly significant positive correlation with *JUN* (ρ = 0.208, *p* = 4.35 × 10^−12^).

In the Basal-like subtype, *ACHE* expression showed a significant positive correlation with *JUN* (ρ = 0.184, *p* = 0.012). No statistically significant correlation was observed between *ACHE* and *STAT3*. The HER2-enriched subtype displayed a complete decoupling of *ACHE* from the evaluated transcriptional network. No significant correlations were found between *ACHE* expression and *STAT3* or *JUN*. In the Luminal A subtype, *ACHE* expression demonstrated a strong and highly significant positive correlation with *JUN* (ρ = 0.255, *p* = 9.25 × 10^−10^). The correlation with *STAT3* showed a weak negative trend but did not reach statistical significance (ρ = −0.081, *p* = 0.056). In the Luminal B subtype, *ACHE* expression was uncoupled from both network components. No significant correlations were detected with *STAT3* or *JUN*.

The correlation analysis for *PRLR* shows a different pattern of associations with *STAT3* and *JUN* compared with *ACHE*, with predominantly positive correlations observed for *STAT3* across the analyzed molecular subtypes (Figure 9B). In the overall breast cancer cohort, *PRLR* expression exhibited a remarkably strong and highly significant positive correlation with *STAT3* (ρ = 0.431, *p* = 2.45 × 10^−50^). Conversely, no statistically significant correlation was observed between *PRLR* and *JUN*.

In the Basal-like subtype, *PRLR* expression maintained a strong positive correlation with *STAT3* (ρ = 0.418, *p* = 2.30 × 10^−9^). Additionally, a significant negative correlation was observed with *JUN* (ρ = −0.156, *p* = 0.032). The HER2-enriched subtype displayed the strongest association within the network, with *PRLR* expression showing a positive correlation with *STAT3* (ρ = 0.589, *p* = 6.07 × 10^−9^); however, no significant correlation was found with *JUN*. In the Luminal A subtype, *PRLR* expression demonstrated a highly significant positive correlation with *STAT3* (ρ = 0.461, *p* = 9.58 × 10^−31^), whereas the correlation with *JUN* was non-significant. In the Luminal B subtype, *PRLR* expression was significantly and positively correlated with *STAT3* (ρ = 0.403, *p* = 7.66 × 10^−10^). No significant correlation was detected with *JUN*.

The mRNA expression levels of *STAT3* and *JUN* were compared between breast tumor and matched normal adjacent tissues to assess differences in the expression of these selected genes related to STAT3/JUN signaling (Figure 9C). As shown in Figure 9C, no statistically significant difference in *STAT3* expression was observed between tumor and normal tissues. In contrast, *JUN* expression was significantly higher in normal breast tissues compared to tumor tissues (*p* < 0.001).

The associations between *STAT3* and *JUN* expression and overall survival in breast cancer patients were evaluated using Kaplan–Meier survival analyses (Figure 9D). Patients were stratified into low (black line) and high (red line) expression groups based on median transcript levels. High expression of *STAT3* was associated with a higher survival probability over time, indicating a trend toward improved overall survival, although this association did not reach statistical significance (HR = 0.84, 95% CI: 0.64–1.09, log-rank *p* = 0.19). Similarly, the expression levels of *JUN* did not show a statistically significant association with patient overall survival, exhibiting a neutral prognostic profile.

### 2.10. Integrated Summary of Signaling Responses Across Breast Cancer Subtypes

A schematic summary of the predominant statistically significant phosphoprotein changes induced by ACh, PRL, and their combination in the MCF7, T47D, and MDA-MB-231 cell lines is presented in Figure 10.

A summary of the transcriptomic profiling of signaling-related genes in breast cancer can be seen in Figure 11, including correlation analyses, differential expression in tumor versus normal tissues, and the impact of gene expression on overall survival.

## 3. Discussion

Breast cancer is a heterogeneous disease in which tumor behavior is determined by the interaction of endocrine signals, growth-factor receptors, intracellular kinases, transcriptional regulators, and the tumor microenvironment [1,2,17,18,19,20,21]. The biological and clinical differences among luminal A, luminal B, HER2-enriched, and basal/triple-negative tumors reflect distinct molecular programs and signaling dependencies [17,18,19,20,21].

Within this complex regulatory environment, ACh and PRL may represent additional factors influencing breast cancer biology. ACh is classically recognized as a neurotransmitter, but cholinergic components are also present in non-neuronal tissues and tumors. Previous studies have linked ACh-associated signaling with breast cancer cell proliferation, angiogenesis, ERK activation, and estrogen receptor regulation [27,28,29]. PRL is a mammary hormone and cytokine that signals through the PRLR and can engage JAK/STAT, PI3K/AKT, MAPK, Src, and focal-adhesion-associated signaling [12,14,16,26,30,31].

The present study combines experimental phosphoprotein data from MCF7, T47D, and MDA-MB-231 breast cancer cell lines with transcriptomic and clinical data from breast cancer cohorts. The experimental analysis assessed changes in selected phosphoproteins after ACh, PRL, or combined ACh + PRL treatment. The transcriptomic analysis evaluated associations between *ACHE* or *PRLR* expression and genes encoding selected signaling proteins, in addition to tumor-versus-normal expression and overall-survival associations.

These analyses evaluate different molecular levels. The experimental data reflect ligand-responsive changes in protein phosphorylation under controlled in vitro conditions, whereas the transcriptomic data reflect gene-expression relationships in human breast tumors. Accordingly, the results should be interpreted as complementary evidence of ACh- and PRL-associated molecular regulation rather than as direct proof of complete pathway activation or causality.

The main experimental finding was that ACh and PRL produced cell-line-specific phosphorylation responses. The combined treatment did not simply reproduce the effect of either ligand alone, indicating that simultaneous exposure may redistribute signaling among parallel regulatory branches.

In the MCF7 cell line, ACh increased phosphorylation of AKT1/2/3, p70S6K, and p53, while reducing phosphorylation of EGFR, ERK1/2, PLC-γ1, and Yes1. PRL produced a similar reduction in several EGFR-, ERK-, PLC-γ1-, and Yes1-associated signals. In contrast, combined ACh + PRL increased AKT phosphorylation at S473/T308 and STAT3 S727 phosphorylation. This pattern is compatible with preferential engagement of AKT- and STAT3-associated signaling after combined stimulation, despite attenuation of selected EGFR/MAPK- and Src-family-associated signals.

The increase in AKT and p70S6K phosphorylation may reflect signaling related to survival, protein synthesis, and growth control. p70S6K is encoded by *RPS6KB1*, and the T421/S424 signal should be assigned to p70S6K1 when the antibody or phosphoproteomic annotation supports this assignment. However, the observed phosphorylation changes should not be interpreted as evidence that the entire AKT/mTOR pathway was activated, because only selected proteins and phosphosites were measured.

The T47D cell line displayed a different response. ACh reduced AKT1/2/3 T308, Yes1 Y426, and STAT3 S727 phosphorylation, whereas PRL increased p53 S46 and decreased RSK1/2/3 phosphorylation. Combined ACh + PRL increased p70S6K1, PLC-γ1, and Src phosphorylation but reduced AKT S473, ERK1/2, and c-Jun phosphorylation. This mixed profile suggests selective signal redistribution rather than uniform activation. Increased Src and PLC-γ1 phosphorylation in the presence of reduced ERK and c-Jun phosphorylation may reflect feedback regulation, temporal differences between proximal and distal signaling, or pathway-specific adaptation.

The MDA-MB-231 cell line showed the broadest activating response. ACh increased RSK1/2/3, ERK1/2, PLC-γ1, Src, and Yes1 phosphorylation. PRL increased AKT1/2/3 T308, EGFR Y1086, and p53 S46 phosphorylation. Combined ACh + PRL increased AKT1/2/3 T308, RSK1/2/3, STAT3 S727, and c-Jun S63 phosphorylation. This coordinated response suggests engagement of survival-, growth-, kinase-, and transcription-associated signaling in this basal/triple-negative model.

The differences among MCF7, T47D, and MDA-MB-231 cell lines indicate that ACh- and PRL-associated responses depend on the pre-existing molecular state of the tumor cell. Hormone-receptor status, PRLR expression, growth-factor signaling, kinase activity, feedback regulation, and transcriptional state may all influence the direction and magnitude of the response.

The transcriptomic analysis identified associations between *ACHE* or *PRLR* expression and genes encoding proteins represented in the experimental analysis or belonging to related signaling groups. These associations varied across molecular subtypes, supporting the concept that the relationship between ACh- and PRL-associated biology and downstream signaling is context-dependent.

The most evident pattern involved AKT–S6 kinase-related genes. *PRLR* was positively associated with several AKT and RSK-family genes across multiple breast cancer subtypes, including *AKT1*, *AKT2*, *AKT3*, *RPS6KB1*, *RPS6KA3*, and *RPS6KA2*. *ACHE* was also positively associated with selected AKT and RSK genes, although these associations were more restricted to specific subtypes. This pattern is compatible with the recognized capacity of *PRLR* to engage PI3K/AKT- and MAPK-associated signaling [12,14,26,31].

The transcriptomic findings provide context for the experimental changes involving AKT, p70S6K, and RSK phosphorylation. However, the association of *PRLR* or *ACHE* with these transcripts does not demonstrate that PRL or ACh directly activated the corresponding proteins. Transcript abundance and phosphorylation represent distinct biological processes.

The EGFR–ERK-related genes showed a similarly context-dependent pattern. *ACHE* was positively associated with *EGFR* in several subtypes and with *MAPK3* in HER2 tumors. *PRLR* showed positive associations with *MAPK1* and *MAPK3* in several cohorts, while its association with *EGFR* differed between the overall cohort and individual subtypes. These findings are compatible with PRLR–EGFR/HER2 crosstalk and with the capacity of ACh-associated signaling to influence ERK-related responses [26,27,32]. Nevertheless, the experimental results showed that EGFR and ERK phosphorylation could increase or decrease depending on the cell line and treatment.

The TP53–Src/PLC-γ1-related genes provided another point of correspondence. *ACHE* and *PRLR* were associated with *PLCG1*, *SRC*, and/or *YES1* in several tumor subtypes. Experimentally, ACh increased PLC-γ1, Src, and Yes1 phosphorylation in the MDA-MB-231 cell line, while combined ACh + PRL increased PLC-γ1 and Src phosphorylation in the T47D cell line. These observations suggest that Src-family and PLC-γ1-associated signaling may represent points of interaction between ACh- and PRL-related biology. This interpretation is also consistent with evidence that PRL can stimulate Src-dependent signaling in breast cancer cells [30].

The STAT3–JUN-related genes showed the greatest context dependence. *PRLR* was positively associated with *STAT3* across most major subtypes, whereas *ACHE* showed a negative association with *STAT3* in the overall cohort. *JUN* was positively associated with *ACHE* in the overall, basal, and Luminal A groups but negatively associated with *PRLR* in basal tumors. In the experimental data, combined ACh + PRL increased STAT3 and c-Jun phosphorylation in the MDA-MB-231 cell line but reduced c-Jun phosphorylation in the T47D cell line. Together, these findings suggest context-dependent regulation of STAT3- and JUN-associated signaling rather than a single linear response.

The tumor-versus-normal and survival analyses further demonstrated that individual genes within the same signaling group can show different expression patterns and clinical associations. Some AKT and RSK-family genes were higher in tumors, whereas others were higher in normal tissues. Similarly, *MAPK3* was associated with improved overall survival, whereas *MAPK1* was associated with poorer overall survival. These results emphasize that gene paralogs should not be assumed to have equivalent biological or prognostic functions.

The relationship between experimental and transcriptomic data is best understood as convergence across molecular levels. For instance, in the AKT–S6 kinase-related group, the experimental data showed ligand-dependent changes in AKT, p70S6K, and RSK phosphorylation. The transcriptomic data showed associations of *ACHE* and *PRLR* with selected *AKT*, *RPS6KB1*, and *RPS6KA* genes. Together, these findings suggest that ACh- and PRL-associated biology may intersect with AKT–S6 kinase-related regulatory programs.

In relation to the EGFR–ERK-related group, the experimental data showed that ACh and PRL altered EGFR and ERK1/2 phosphorylation, with the direction depending on the cell line. The transcriptomic data showed associations involving *EGFR*, *MAPK1*, and *MAPK3* in selected cohorts. These findings implicate EGFR–ERK-related signaling as a potential point of convergence but do not establish a uniform direction of pathway activation.

According to the TP53–Src/PLC-γ1-related group, the experimental data showed treatment-associated changes in p53, PLC-γ1, Src, and Yes1 phosphorylation. The transcriptomic data showed tumor-level associations involving *TP53*, *PLCG1*, *SRC*, and *YES1*. This overlap suggests that ACh- and PRL-associated signals may intersect with Src-family and PLC-γ1-related regulatory networks.

In the STAT3–JUN-related group, the experimental data showed cell-line-specific changes in STAT3 and c-Jun phosphorylation, particularly after combined treatment. The transcriptomic data showed broad *PRLR*–*STAT3* associations but subtype-dependent relationships involving *JUN*. These findings suggest that transcription-associated responses to ACh and PRL are strongly dependent on tumor-cell context.

This integrated interpretation should remain cautious. The experimental data measure ligand-responsive phosphorylation, whereas the transcriptomic data measure gene-expression associations and clinical relationships in breast cancer cohorts. Thus, the overlap identifies biologically plausible points of convergence but does not constitute direct molecular validation.

The results support a model in which ACh and PRL may influence overlapping regulatory networks in breast cancer. PRLR-associated signaling can involve JAK/STAT, PI3K/AKT, MAPK, Src, and EGFR/HER2-associated crosstalk [12,14,26,30,31]. ACh-associated signaling has also been linked to PLC, Src, ERK, and PI3K/AKT-related processes in cancer models [27,28,29,32]. These shared effectors provide a plausible basis for the combined-treatment responses observed in the present study.

However, the data do not demonstrate that ACh and PRL act synergistically. The combined treatment produced both increased and decreased phosphorylation events, depending on the cell line and protein examined. The term “crosstalk” should therefore be used as a hypothesis describing possible interaction between signaling inputs, not as proof of a defined molecular mechanism.

### Limitations of This Study

Several limitations should be acknowledged. (i) The phospho-kinase array is a semiquantitative, membrane-based immunoassay; the changes reported here have not been confirmed by an orthogonal method such as Western blotting or phospho-specific ELISA, which reviewers of this type of study will normally expect for the principal targets. (ii) A single time point (4 h) and a single concentration of each agonist were examined; kinase phosphorylation is highly dynamic, and both earlier and later responses may have been missed. (iii) Thirty-seven targets were screened in three cell lines under four conditions without correction for multiple comparisons, so individual *p*-values close to 0.05 should be regarded as exploratory. (iv) The capture antibody does not discriminate among AKT isoforms, limiting the integration of the array data with the isoform-level cohort analysis. (v) The in vitro and in silico datasets measure different molecular quantities, protein phosphorylation and mRNA abundance, and are therefore complementary rather than mutually confirmatory; no causal inference should be drawn from their juxtaposition. (vi) Only three established cell lines were used, two of which are luminal, and no non-tumorigenic mammary epithelial control (for example, MCF-10A) was included. (vii) Receptor dependence was not established; no muscarinic, nicotinic, or PRLR antagonist was used, so the observed responses cannot be formally attributed to a specific receptor.

## 4. Materials and Methods

### 4.1. Cell Culture

The MCF7 (ATCC HTB-22, #C0006008), T47D (ATCC HTB-133, #C0006001), and MDA-MB-231 (ATCC HTB-26, #C0006002) (Addexbio, San Diego, CA, USA) human breast cancer cell lines were cultured as follows: MCF7 cell lines were grown in Dulbecco’s Modified Eagle’s Medium (DMEM; Gibco, Carlsbad, CA, USA) supplemented with 10 µg/mL insulin (sc-360248, Santa Cruz Biotechnology, Santa Cruz, CA, USA) and 10% fetal bovine serum (FBS; HyClone, Fremont, CA, USA). The T47D cell line was maintained in RPMI-1640 (HyClone, Logan, UT, USA) with 10% FBS. The MDA-MB-231 cell line was cultured in high-glucose DMEM (4500 mg/L; Gibco) with 10% FBS. For all experiments, the cells were transferred 48 h before treatment to hormone-reduced medium consisting of phenol red-free DMEM (Gibco) supplemented with 5% charcoal-stripped FBS (Gibco), 1% sodium pyruvate, and 1% L-glutamine. All media contained penicillin (100 U/mL) and streptomycin (100 µg/mL; Gibco). Cells were grown in 75 cm^2^ flasks (Corning, Tewksbury, MA, USA) at 37 °C in a humidified 5% CO_2_ atmosphere. Subculture was performed with 0.05% trypsin-EDTA upon reaching 80% confluence. MCF7 and T47D were used as ERα-positive luminal models [17,19,33] and MDA-MB-231 as a triple-negative model [34].

### 4.2. Chemical Reagents

Acetylcholine chloride (purity ≥ 99%, Sigma-Aldrich, St. Louis, MO, USA) was dissolved in sterile water, filtered through a 0.22 µm membrane, and stored as a 1 × 10^−3^ M stock at 4 °C in the dark. The working concentration was 1 × 10^−7^ M (previously reported by the authors). Human recombinant prolactin (Sigma-Aldrich, Cat. No. L6520) was dissolved in sterile PBS at 25 µg/mL and stored at −20 °C and used at a final concentration of 100 µg/mL.

### 4.3. Phosphoprotein Array Assay

Phosphoprotein profiling was performed using the Proteome Profiler™ Human Phospho-Kinase Array Kit (R&D Systems, Cat. No. ARY003C; Bio-Techne, Minneapolis, MN, USA), which detects the relative phosphorylation of 37 kinase phosphorylation sites and 2 total proteins on duplicate nitrocellulose membranes, following the manufacturer’s instructions. Briefly, MCF7, T47D, and MDA-MB-231 cell lines were grown to ~80% confluence in 100 mm dishes, hormone-deprived for 48 h, and treated with vehicle control, ACh (1 × 10^−7^ M), PRL (100 µg/mL), or ACh + PRL for 4 h. Cells were lysed in the kit-supplied Lysis Buffer 6 containing protease and phosphatase inhibitors, and protein concentration was determined by BCA assay. Equal amounts of total protein (300 µg) were diluted in Array Buffer 1 and incubated overnight at 4 °C on nitrocellulose membranes spotted in duplicate with capture antibodies. After washing with Wash Buffer, membranes were sequentially incubated with the kit-supplied biotinylated detection antibody cocktail and streptavidin-HRP, developed with Chemi Reagent Mix, and imaged on a C-DiGit scanner (LI-COR Biosciences, Lincoln, NE, USA). Spot pixel densities were quantified using ImageJ 1.53t (NIH) and normalized to the average signal of the reference (positive control) spots on each membrane to allow inter-membrane comparison. From the complete 37-site Proteome Profiler Human Phospho-Kinase Array, a targeted panel of phosphorylation sites was selected to provide a pathway-oriented assessment of cellular responses to ACh, PRL, and their combination. The panel included AKT1/2/3 at T308 and S473, p70S6K at T421/S424, RSK1/2/3 at S380/S386/S377, EGFR at Y1086, ERK1/2 at T202/Y204 and T185/Y187, p53 at S46, PLC-γ1 at Y783, Src (SRC) at Y419, Yes (YES1) at Y426, STAT3 at S727, and c-Jun at S63. These analytes were selected to represent receptor-proximal signaling, PI3K/AKT- and mTOR-associated pathways, MAPK/ERK and Src-family kinase activity, downstream transcriptional effectors, and cellular stress responses. Because the assay detects relative phosphorylation at predefined epitopes, the results were interpreted as treatment-associated changes in signaling-state markers rather than as direct measurements of total protein abundance or catalytic kinase activity [24]. The array detects total (pan) phosphorylation of AKT1/2/3 and does not distinguish among the individual AKT isoforms.

### 4.4. Bioinformatic Analyses of Public Breast Cancer Cohorts

Gene symbols were standardized throughout the manuscript using the official human gene symbols approved by the HUGO Gene Nomenclature Committee (HGNC, RRID: SCR_002827). Corresponding protein names were provided where appropriate. Specifically, *RPS6KB1* encodes p70S6K (p70S6K1); *RPS6KA1*, *RPS6KA3*, and *RPS6KA2* encode RSK1, RSK2, and RSK3, respectively; *MAPK1* and *MAPK3* encode ERK2 and ERK1, respectively; and *PLCG1* encodes PLC-γ1. Correlations between *ACHE* or *PRLR* and each target gene, and the comparison of transcript levels between breast tumor and normal tissue, were obtained using the Tumor Immune Estimation Resource version 3 (TIMER3, https://compbio.cn/timer3/, accessed on 7 July 2026) [35], using the TCGA breast invasive carcinoma cohort (*n* = 1086). Correlations were assessed using Spearman’s rank correlation coefficient (ρ), whereas tumor-versus-normal comparisons were computed by the platform using edgeR based on RNA-Seq raw count data of the Gene_DE module of TIMER3.0. Overall survival analyses were retrieved from the Kaplan–Meier Plotter (https://www.kmplot.com/analysis/, accessed on 13 July 2026) [36], using the breast cancer mRNA gene chip module of Kaplan–Meier Plotter. The platform supports probe-set analysis based on the Affymetrix GeneChip Human Genome U133 Plus 2.0 Array (HG-U133 Plus 2.0; GEO platform GPL570), with patients dichotomized by the platform’s automatically selected best cut-off; hazard ratios with 95% confidence intervals and log-rank *p*-values were reported as returned by the platform. Unless otherwise specified, platform default settings were used. No additional multiple-testing correction was applied across the set of analyses performed in this study. Given the exploratory and multi-target nature of the array-based screening, results with *p*-values close to the 0.05 threshold should be interpreted as hypothesis-generating rather than confirmatory.

### 4.5. Statistical Analysis

Statistical analyses were performed using GraphPad Prism version 5.0 (GraphPad Software, Inc., La Jolla, CA, USA). For comparisons involving more than two groups, data were analyzed using the Kruskal–Wallis non-parametric test, followed by Dunn’s multiple-comparisons test for post hoc pairwise comparisons, as appropriate. Comparisons were performed between the untreated control group and the respective treated groups. Statistical significance was defined as *p* < 0.05. At least three independent experiments were performed for each experimental condition. Numerical data are presented as the mean ± standard error of the mean (SEM).

## 5. Conclusions

The findings indicate that ACh and PRL are additional factors associated with breast cancer signaling. ACh, PRL, and combined ACh + PRL stimulation produced cell-line-specific phosphorylation responses, while *ACHE* and *PRLR* expression showed subtype-dependent associations with genes encoding proteins within AKT–S6 kinase-, EGFR–ERK-, TP53–Src/PLC-γ1-, and STAT3–JUN-related signaling groups.

The experimental and transcriptomic findings converge on related molecular modules but represent different biological levels. The experimental data reflect ligand-responsive phosphorylation, whereas the transcriptomic data reflect tumor-level gene-expression associations and clinical relationships. Together, they support a model in which ACh- and PRL-associated signals may intersect with established breast cancer regulatory networks, while emphasizing that these interactions are context-dependent and require functional validation.

## Figures and Tables

**Figure 1:**
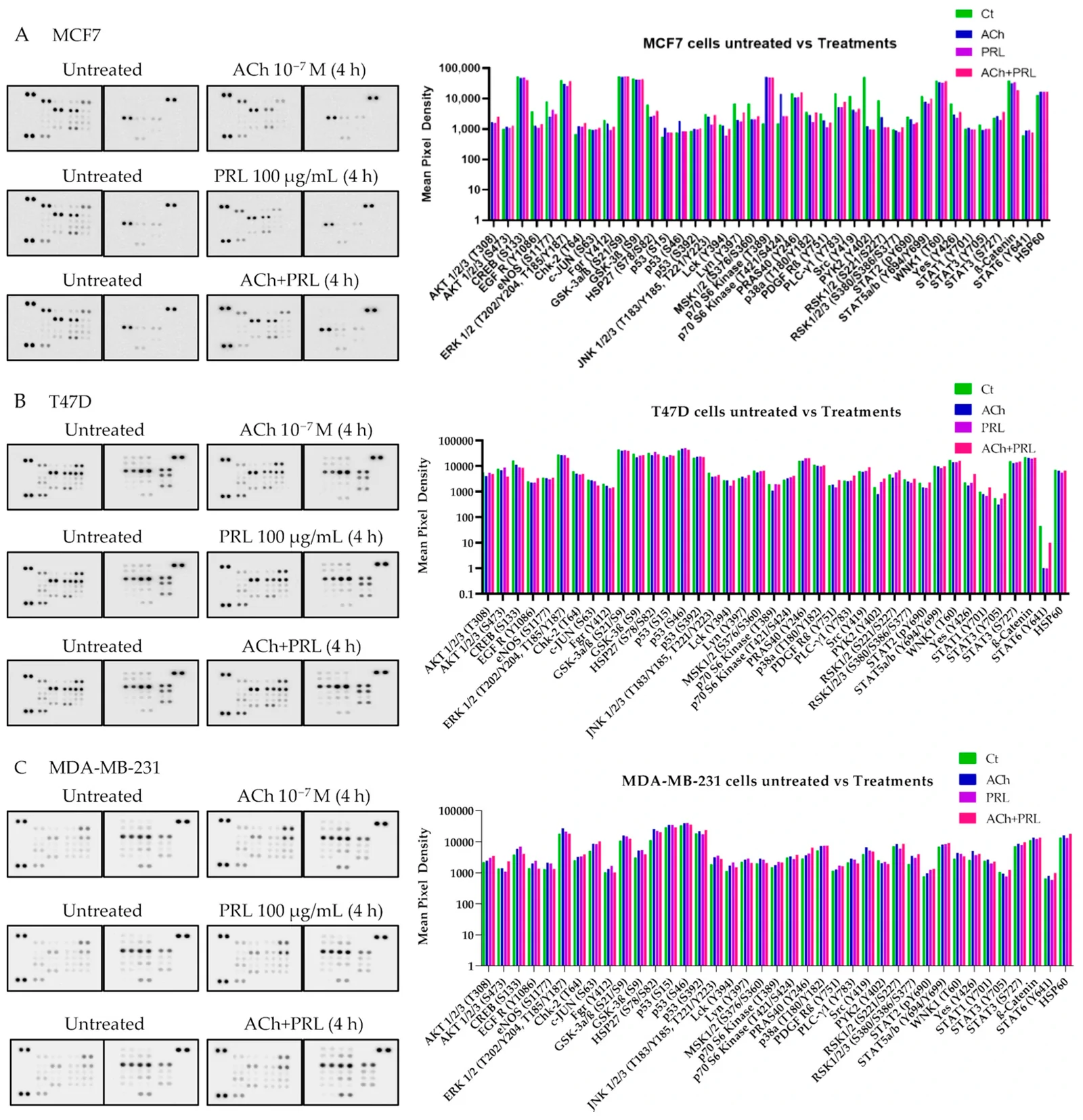
Global kinase phosphorylation profiles in breast cancer cell lines treated with ACh and PRL. Phospho-kinase profiling was conducted on (**A**) MCF7 (ER+), (**B**) T47D (ER+), and (**C**) MDA-MB-231 (TNBC) cell lines using the Proteome Profiler™ Human Phospho-Kinase Array Kit. The data illustrate changes in protein phosphorylation levels at various kinase targets following treatment with $10^{-7}$ M acetylcholine (ACh) or 100 µg/mL prolactin (PRL), compared to untreated controls, after 4 h of incubation. Panels (**A**–**C**) also show representative spot blots quantifying the phosphorylation signal for selected targets across all tested conditions. Bar graphs represent the mean pixel density, providing relative quantitative levels of phosphorylation (p-kinase) normalized against the untreated control.

**Figure 3:**
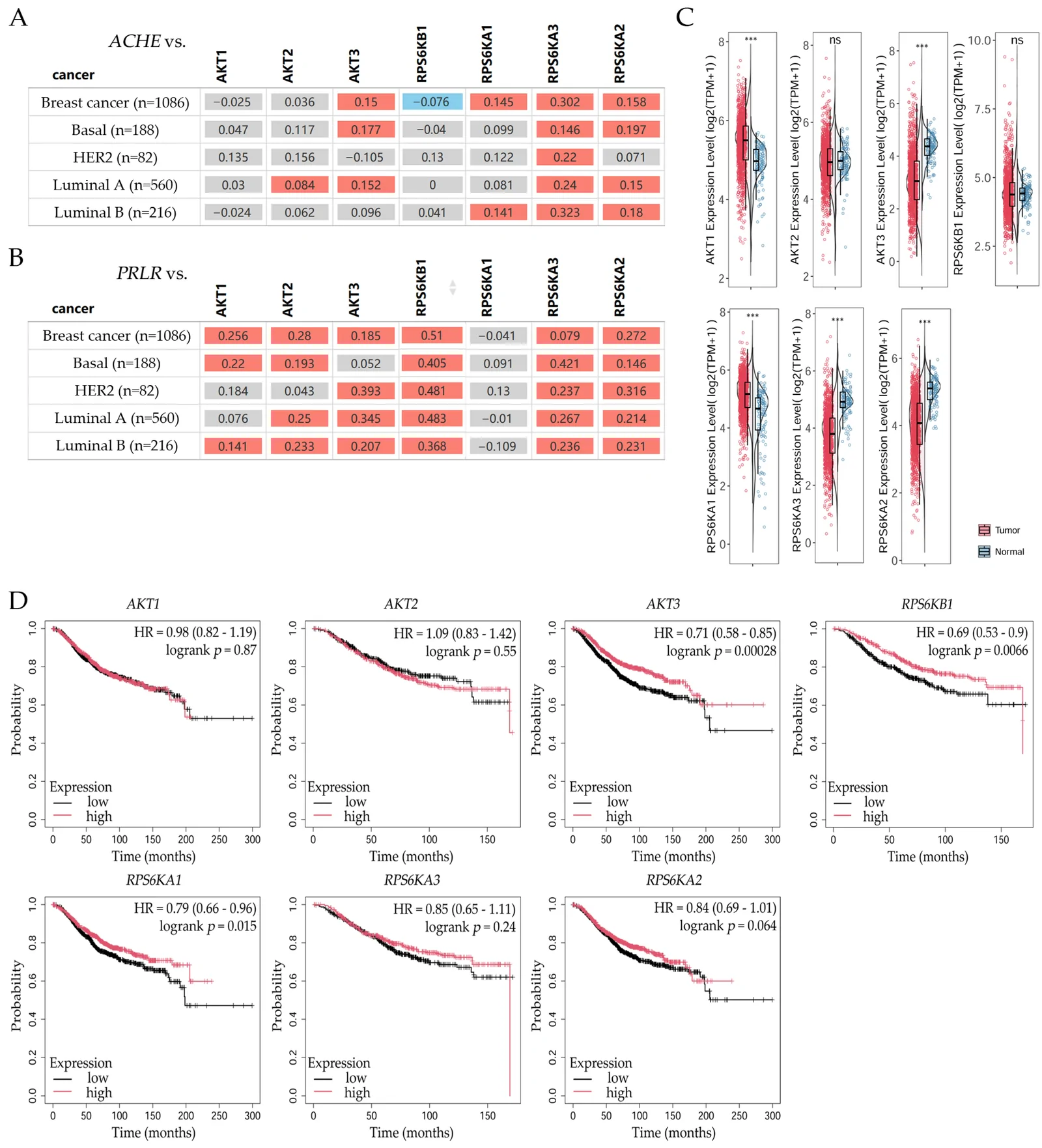
(**A**,**B**) Correlation analysis of acetylcholinesterase (*ACHE*) and prolactin receptor (*PRLR*) with AKT/mTOR signaling genes in breast cancer. Correlation heatmap displaying Spearman correlation coefficients (ρ) between *ACHE* (Panel **A**) or *PRLR* (Panel **B**) expression and the expression of AKT and RPS6K family genes, specifically AKT serine/threonine kinase 1 (*AKT1*), AKT serine/threonine kinase 2 (*AKT2*), AKT serine/threonine kinase 3 (*AKT3*), ribosomal protein S6 kinase B1 (*RPS6KB1*), ribosomal protein S6 kinase A1 (*RPS6KA1*), ribosomal protein S6 kinase A3 (*RPS6KA3*), and ribosomal protein S6 kinase A2 (*RPS6KA2*), across overall breast cancer and its molecular subtypes (Basal, HER2, Luminal A, and Luminal B). Red indicates statistically significant positive correlations (Spearman’s rank correlation, *p* < 0.05), blue indicates statistically significant negative correlations (*p* < 0.05), and gray represents non-significant associations. Data were obtained from the Tumor Immune Estimation Resource v3.0 (TIMER3.0), accessed on 7 July 2026. (**C**) Jitter plots depicting differential expression analysis of *AKT* and *RPS6K* family genes (*AKT1*, *AKT2*, *AKT3*, *RPS6KB1*, *RPS6KA1*, *RPS6KA3*, and *RPS6KA2*) in breast tumor (red) and normal (blue) tissues. Gene expression levels are represented as log2(TPM + 1). Statistical significance was computed by differential analysis (edgeRassessed by TIMER3.0, https://compbio.cn/timer3/, accessed on 7 July 2026) on RNA-Seq raw counts and annotated by the number of stars (***: *p*-value < 0.001), ns: non-significant. Data were extracted from TIMER3.0, accessed on 7 July 2026. (**D**) Kaplan–Meier survival curves evaluating the prognostic value of *AKT1*, *AKT2*, *AKT3*, *RPS6KB1*, *RPS6KA1*, *RPS6KA3*, and *RPS6KA2* gene expression in breast cancer patients. Patients were categorized into high (red line) and low (black line) expression groups based on the median expression levels. The *x*-axis shows time in months, and the *y*-axis indicates the probability of survival. The hazard ratio (HR) with 95% confidence intervals and the log-rank *p*-value are provided for each gene. Analysis obtained from the Kaplan–Meier Plotter(https://www.kmplot.com/analysis/), accessed 13 July 2026.

**Figure 5:**
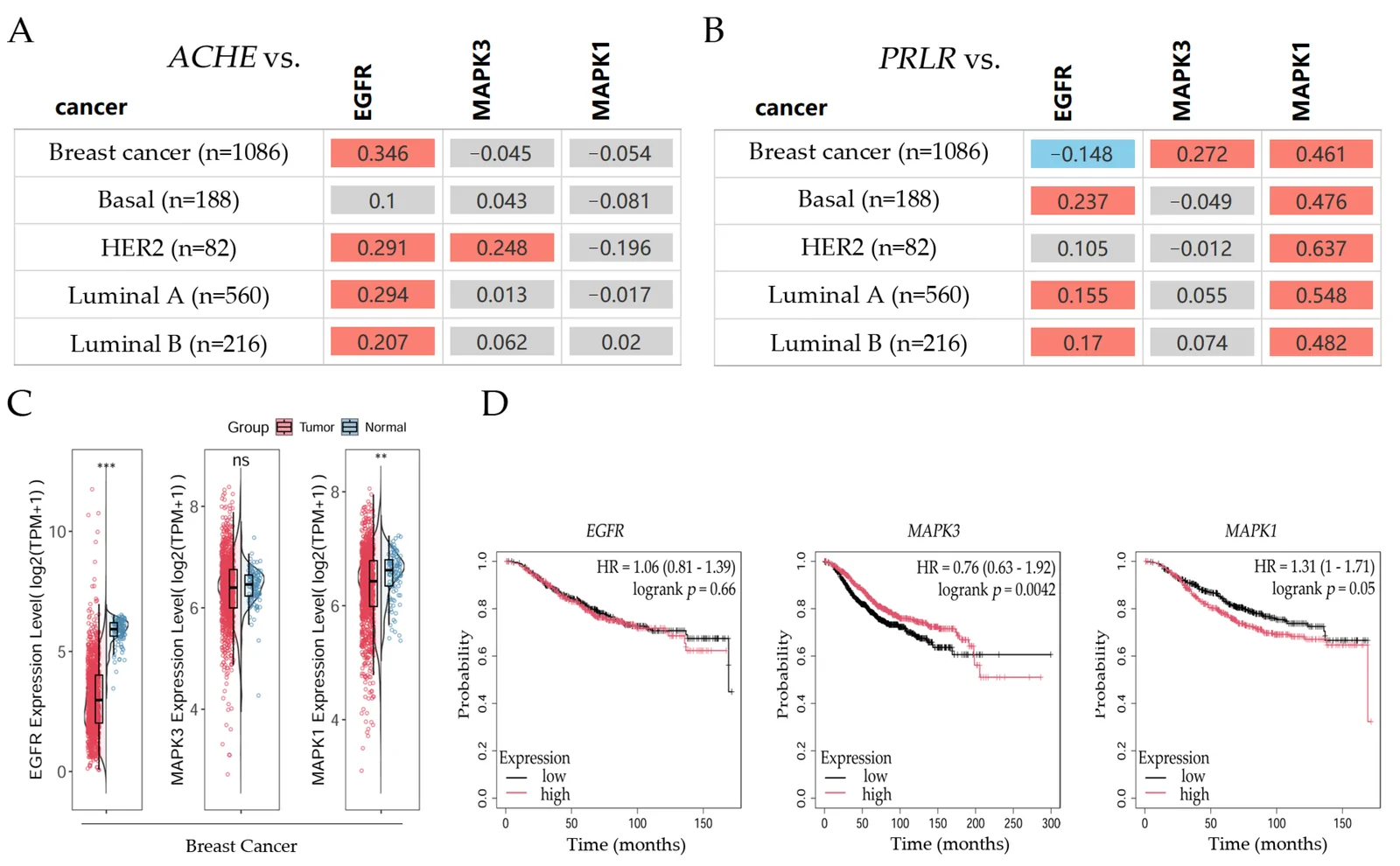
(**A**,**B**) Association of acetylcholinesterase (*ACHE*) and prolactin receptor (*PRLR*) with the EGFR/MAPK signaling pathway in breast cancer. Correlation heatmap displaying Spearman correlation coefficients (ρ) between *ACHE* (Panel **A**) or *PRLR* (Panel **B**) expression and components of the EGFR/MAPK cascade, namely epidermal growth factor receptor (*EGFR*), mitogen-activated protein kinase 3 (*MAPK3*), and mitogen-activated protein kinase 1 (*MAPK1*), across overall breast cancer and its molecular subtypes (Basal, HER2, Luminal A, and Luminal B). Red indicates statistically significant positive correlations (Spearman’s rank correlation, *p* < 0.05), blue indicates statistically significant negative correlations (*p* < 0.05), and gray represents non-significant associations. Data were obtained from the Tumor Immune Estimation Resource v3.0 (TIMER3.0), accessed on 7 July 2026. (**C**) Jitter plot depicting differential expression analysis of *EGFR*, *MAPK3*, and *MAPK1* gene expression levels between breast tumor (red) and normal (blue) tissues. Gene expression levels are represented as log2(TPM + 1). Statistical significance was computed by differential analysis (edgeR) on RNA-Seq raw counts and annotated by the number of stars (**: *p*-value < 0.01; ***: *p*-value < 0.001), ns: non-significant. Data extracted from TIMER3.0, accessed on 7 July 2026. (**D**) Kaplan–Meier survival curves showing the association of *EGFR*, *MAPK3*, and *MAPK1* expression in breast cancer patients. Patients were categorized into high (red line) and low (black line) expression groups based on the median expression levels. The *x*-axis shows time in months, and the *y*-axis indicates the probability of survival. The hazard ratio (HR) with 95% confidence intervals and the log-rank *p*-value are provided for each gene. Data obtained from Kaplan–Meier Plotter, accessed 13 July 2026.

**Figure 7:**
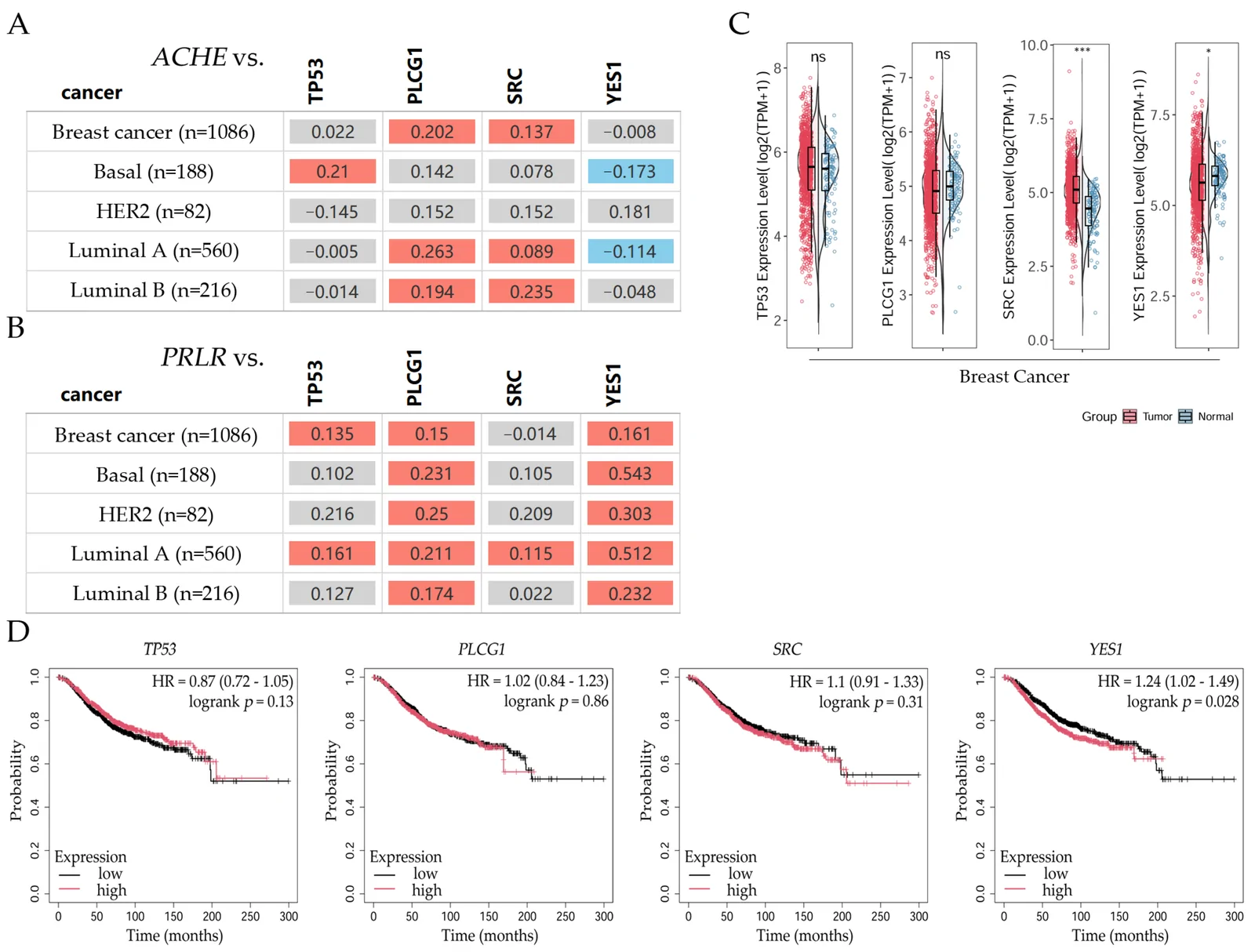
(**A**,**B**) Correlation of acetylcholinesterase (*ACHE*) and prolactin receptor (*PRLR*) with the TP53/SRC signaling network in breast cancer. Correlation heatmaps displaying Spearman correlation coefficients (ρ) between *ACHE* (Panel **A**) or *PRLR* (Panel **B**) expression and key components of the TP53/SRC signaling network, specifically the tumor protein p53 (*TP53*), phospholipase C gamma 1 (*PLCG1*), the SRC proto-oncogene, non-receptor tyrosine kinase (*SRC*), and the YES proto-oncogene 1, Src family tyrosine kinase (*YES1*), across overall breast cancer and its molecular subtypes (Basal, HER2, Luminal A, and Luminal B). Red indicates statistically significant positive correlations (Spearman’s rank correlation, *p* < 0.05), blue indicates statistically significant negative correlations (*p* < 0.05), and gray represents non-significant associations. Data were obtained from the Tumor Immune Estimation Resource v3.0 (TIMER3.0), accessed on 7 July 2026. (**C**) Jitter plot showing differential mRNA expression levels of *TP53*, *PLCG1*, *SRC*, and *YES1* between breast tumor (red) and normal adjacent (blue) tissues. Statistical significance was computed by differential analysis (edgeR) on RNA-Seq raw counts and annotated by the number of stars (*: *p*-value < 0.05; ***: *p*-value < 0.001), ns: non-significant. Data retrieved from TIMER3.0, accessed on 7 July 2026. (**D**) Kaplan–Meier overall survival curves for breast cancer patients stratified by low (black line) and high (red line) expression levels of *TP53*, *PLCG1*, *SRC*, and *YES1*. The hazard ratio (HR) with 95% confidence intervals and the log-rank *p*-value are indicated for each gene. Data obtained from the Kaplan–Meier Plotter, accessed 13 July 2026.

**Figure 9:**
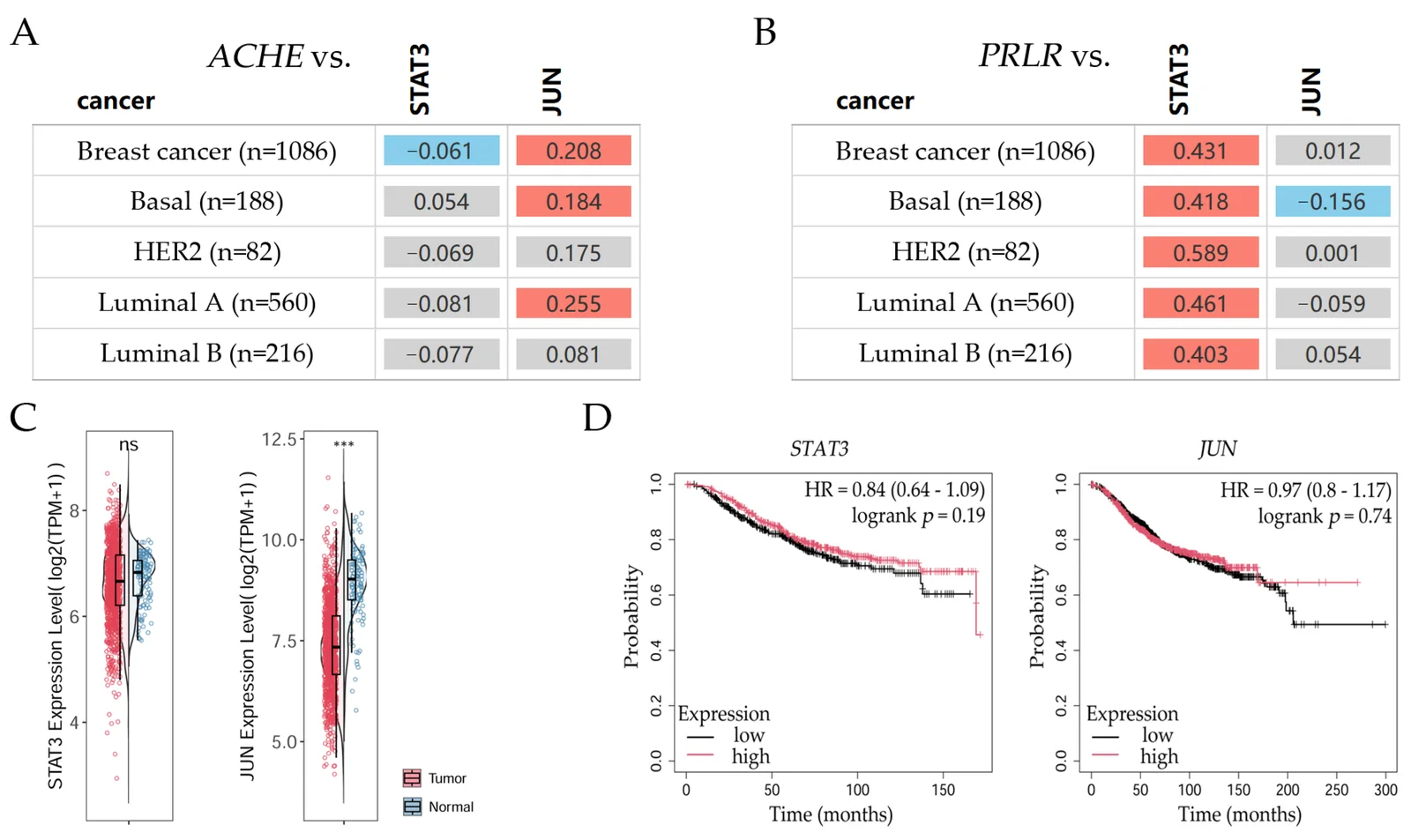
(**A**,**B**) Correlation of acetylcholinesterase (*ACHE*) and prolactin receptor (*PRLR*) expression with key AP-1 and JAK-STAT pathway-associated genes (*JUN* and *STAT3*) in breast cancer. Correlation heatmap displaying Spearman correlation coefficients (ρ) between *ACHE* (Panel **A**) or *PRLR* (Panel **B**) expression and key components of the STAT3/JUN transcriptional network, specifically the signal transducer and activator of transcription 3 (*STAT3*) and the Jun proto-oncogene, AP-1 transcription factor subunit (*JUN*), across overall breast cancer and its molecular subtypes (Basal, HER2, Luminal A, and Luminal B). Red indicates statistically significant positive correlations (Spearman’s rank correlation, *p* < 0.05), blue indicates statistically significant negative correlations (*p* < 0.05), and gray represents non-significant associations. Data were obtained from the Tumor Immune Estimation Resource v3.0 (TIMER3.0), accessed on 7 July 2026. (**C**) Jitter plot depicting differential mRNA expression levels of *STAT3* and *JUN* between breast tumor (red) and normal adjacent (blue) tissues. The analysis was done using edgeR on RNA-Seq raw counts. Statistical significance was annotated by the number of stars (***: *p*-value < 0.001), ns: non-significant. Data retrieved from TIMER3.0, accessed on 7 July 2026. (**D**) Kaplan–Meier overall survival curves for breast cancer patients stratified by low (black line) and high (red line) expression levels of *STAT3* and *JUN*. The hazard ratio (HR) with 95% confidence intervals and the log-rank *p*-value are indicated for each gene. Data were sourced from the Kaplan–Meier Plotter, accessed 13 July 2026.

**Figure 10:**
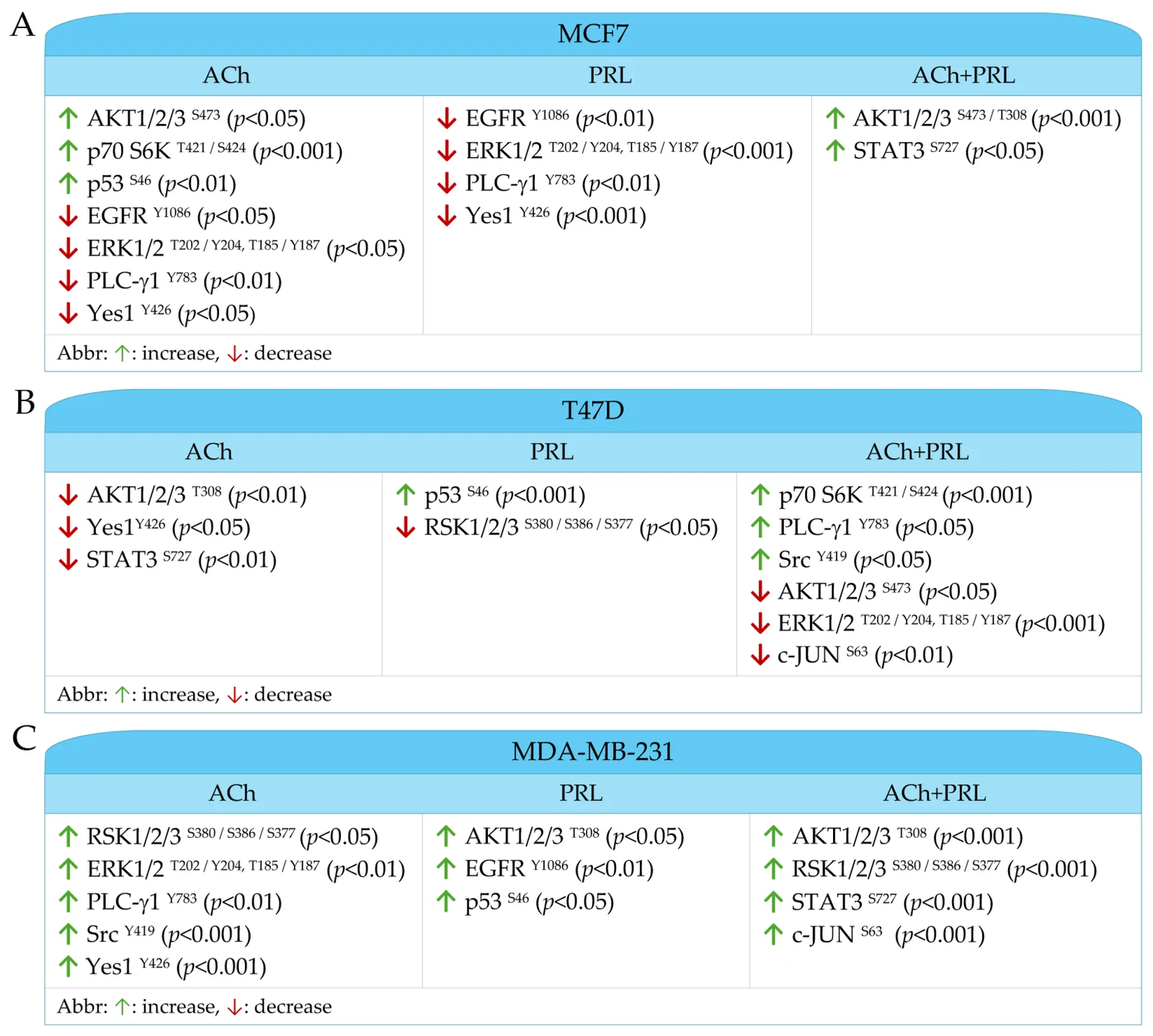
Differential phosphoprotein responses to acetylcholine (ACh), prolactin (PRL), and combined ACh + PRL stimulation in breast cancer cell lines. Summary of significantly altered phosphoproteins identified in (**A**) MCF7, (**B**) T47D, and (**C**) MDA-MB-231 cell lines following 4 h treatment with ACh, PRL, or ACh + PRL. Green arrows indicate significantly increased phosphorylation or protein abundance, whereas red arrows indicate significant decreases relative to untreated controls. Only statistically significant changes are shown. Abbreviations: ACh, acetylcholine; PRL, prolactin; ↑, increase; ↓, decrease.

**Figure 11:**
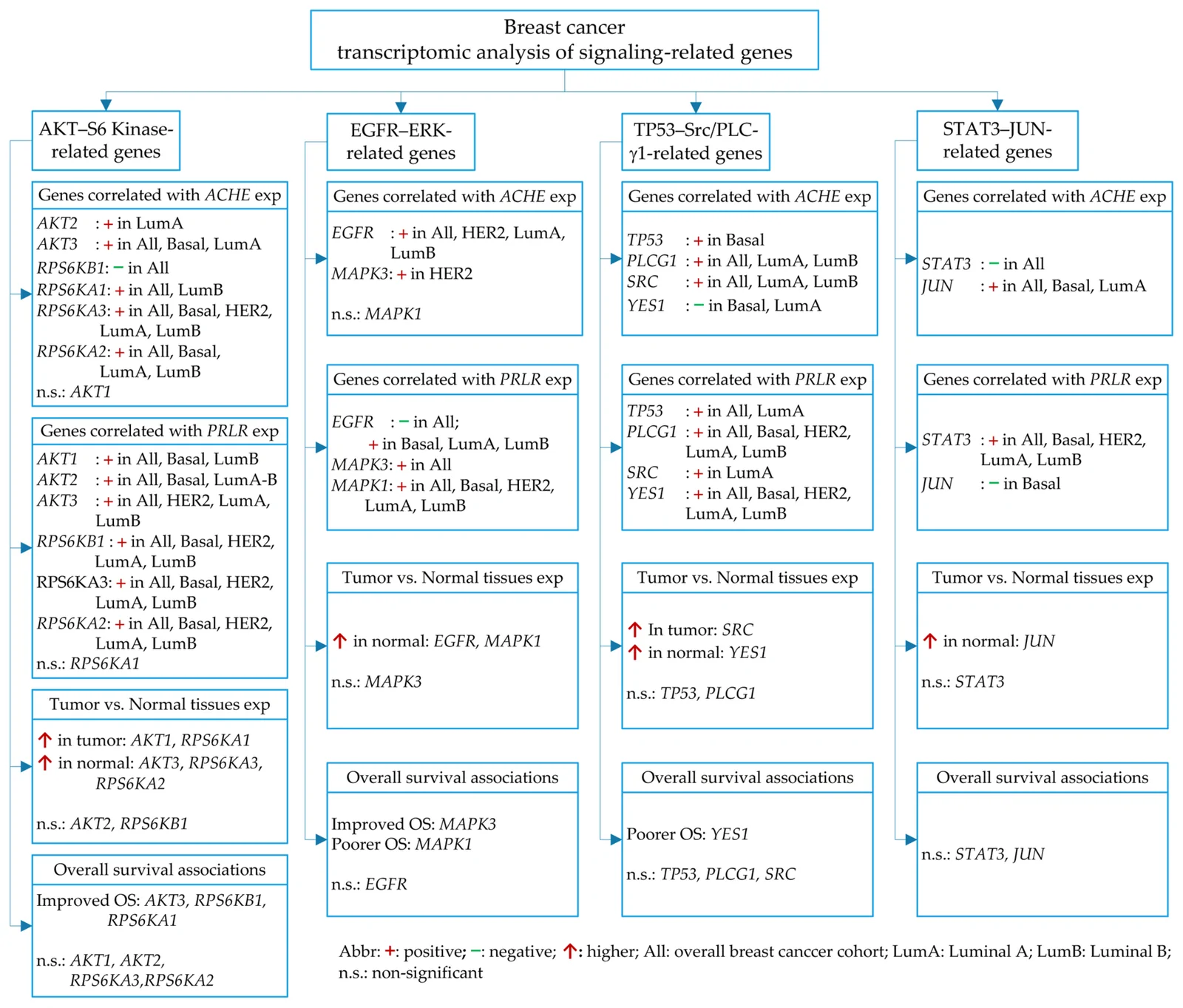
Transcriptomic profiling and clinical associations of signaling-related genes in breast cancer. The data present the correlation of specific signaling genes with *ACHE* and *PRLR* expression across the overall breast cancer cohort and specific molecular subtypes (Basal, HER2, Luminal A, Luminal B). Additionally, the tables summarize differential expression in tumor versus normal tissues and the impact of gene expression on overall survival (OS). Abbreviations: +, positive correlation; −, negative correlation; ↑, higher expression; All, overall breast cancer cohort; LumA, Luminal A; LumB, Luminal B; n.s., non-significant; OS, overall survival.

## Data Availability

The bioinformatic data presented in this study are openly available from the Tumor Immune Estimation Resource v 3.0 (TIMER3.0, http://timer.cistrome.org), accessed on 7 July 2026 [35]; The University of California Santa Cruz (UCSC) Xena Functional Genomics Browser (https://xena.ucsc.edu/), accessed on 10 July 2026 [37]; and from the Kaplan–Meier Plotter (https://www.kmplot.com/analysis/), accessed on 13 July 2026 [36]. Other data generated in the present study may be requested from the corresponding author.

## Abbreviations

The following abbreviations are used in this manuscript:

ACh	Acetylcholine
PRL	Prolactin
qRT-PCR	Quantitative reverse-transcription polymerase chain reaction
ACHE	Acetylcholinesterase
PRLR	Prolactin receptor
ERα	Estrogen receptor-α
OPs	Organophosphorus pesticides
AKT1	AKT serine/threonine kinase 1
AKT2	AKT serine/threonine kinase 2
AKT3	AKT serine/threonine kinase 3
RPS6KB1	Ribosomal protein S6 kinase B1
RPS6KA1	Ribosomal protein S6 kinase A1
RPS6KA3	Ribosomal protein S6 kinase A3
RPS6KA2	Ribosomal protein S6 kinase A2
EGFR	Epidermal growth factor receptor
MAPK3	Mitogen-activated protein kinase 3
MAPK1	Mitogen-activated protein kinase 1
TP53	Tumor protein p53
PLCG1	Phospholipase C gamma 1
SRC	SRC proto-oncogene, non-receptor tyrosine kinase
YES1	YES proto-oncogene 1, Src family tyrosine kinase
STAT3	Signal transducer and activator of transcription 3
JUN	Jun proto-oncogene, AP-1 transcription factor subunit
TIMER3.0	Tumor Immune Estimation Resource v3.0
SEM	Standard error of the mean
